# Biomimetic Synthesis of Resveratrol Trimers Catalyzed by Horseradish Peroxidase

**DOI:** 10.3390/molecules22050819

**Published:** 2017-05-17

**Authors:** Jian-Qiao Zhang, Gan-Peng Li, Yu-Long Kang, Bin-Hao Teng, Chun-Suo Yao

**Affiliations:** 1 State Key Laboratory of Bioactive Substance and Function of Natural Medicines, Institute of Materia Medica, Chinese Academy of Medical Sciences & Peking Union Medical College, Beijing 100050, China; jqzhang@imm.ac.cn (J.-Q.Z.); kangyulongzyy@imm.ac.cn (Y.-L.K.); tengbh@imm.ac.cn (B.-H.T.); 2 Key Laboratory of Chemistry in Ethnic Medicinal Resources, State Ethnic Affairs Commission & Ministry of Education, Yunnan Minzu University, Kunming 650500, China; ganpeng_li@sina.com

**Keywords:** horseradish peroxidase, ε-viniferin, resveratrol trimer, biotransformation, radical reaction

## Abstract

Biotransformation of *trans*-resveratrol and synthetic (±)-ε-viniferin in aqueous acetone using horseradish peroxidase and hydrogen peroxide as oxidants resulted in the isolation of two new resveratrol trimers (**3** and **4**), one new resveratrol derivative (**5**) with a dihydrobenzofuran skeleton, together with two known stilbene trimers (**6** and **7**), and six known stilbene dimers (**8**–**13**). Their structures and relative configurations were identified through spectral analysis and possible formation mechanisms were also discussed. Among these oligomers, trimers **6** and **7** were obtained for the first time through direct transformation from resveratrol. Results indicated that this reaction is suitable for the preparation of resveratrol oligomers with a complex structure.

## 1. Introduction

Stilbenes are a class of plant polyphenols that can be divided into two categories, namely, monomeric and oligomeric stilbenes. Resveratrol oligomers possess novel structures and exhibit various biological activities, such as anticarcinogenesis, anti-inflammation, and tyrosinase activity inhibition. These oligomers can be used to treat cancer, AIDS, bacterial infections, and other diseases. Some oligostilbenes exhibit more potent bioactivities than their monomers do [1,2,3].

**Figure 1:**
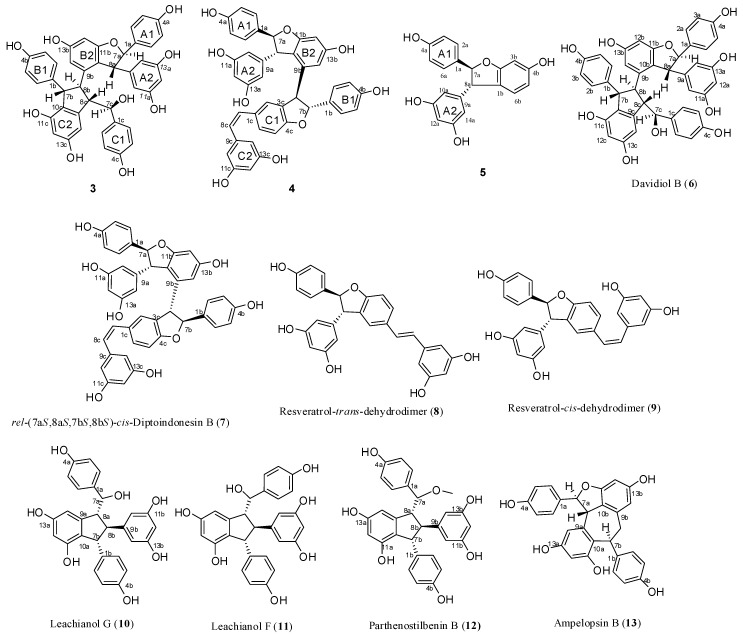
Structures of compounds **3**–**13**.

Various resveratrol oligomers exist in nature, especially in grapevine. However, the structures of minor stilbene oligomers have yet to be elucidated because sufficient amounts of these minor components are difficult to isolate for subsequent structural characterization. In recent years, some researchers focused their attention on the synthesis of these oligomers and total synthetic routes of numerous resveratrol oligomers, including dimers, trimers, and tetramers, have been reported in literature [4,5,6,7,8,9,10], but long reaction steps render these approaches unsuitable for specific preparations of the complex oligomers. Therefore, biomimetic synthesis is still a concise and practical alternative for the preparation of oligostilbenes with intricate structures. The natural biotransformation of stilbene oligomers in nature can be simulated in vitro by transformation with biological enzymes, unorganized fermentation, metal oxidants, light, acids and alkali [2,11]. In combination with diverse separation methods, transformation can induce the accumulation of large amounts of minor compounds. Using this approach, a number of known natural stilbene oligomers and new stilbene oligomers have been obtained, and their structures have also been identified successfully [11]. The oxidative coupling of resveratrol, including its analogs, has been examined under different conditions since ε-viniferin was first isolated in 1977 by Langcake and Pryce [12]. Nevertheless, the in vitro biocatalyzed oxidation of stilbenes has rarely been explored, and studies on biocatalyzed enzymes have focused on horseradish peroxidase (HRP) and laccase [13,14], which indicated that dimers, such as dihydrobenzofuran-like dimers, are the main products of reactions mediated by these enzymes. As reported in the literature [12,15], ε-viniferin, which would be synthesized enzymatically in plant tissues, seems to be a biogenetically important precursor of many oligostilbenes in plants, such as davidiol A, davidiol B, hopeaphenol, ampelopsin E, and so on. So, biotransformation of *ε*-viniferin is a promising way to prepare these oligomers. Wilkens et al. [16] reported the transformation of resveratrol and (−)-ε-viniferin catalyzed by HRP produces different stilbene oligomers, including dimers, trimers, tetramers, and polymeric products. In addition to two trimeric stilbenes obtained in a pure form, other oligomers only have been detected through HPLC-PDA or HPLC-ESI-MS/MS. Many resveratrol trimers and tetramers can be possibly obtained by further investigating this reaction. As an extension of the preliminary work, this study demonstrated the biotransformation of *trans*-resveratrol (**1**) and synthetic (±)-ε-viniferin (**2**) in aqueous acetone with HRP and hydrogen peroxide as oxidants. Thus, two new resveratrol trimers (**3** and **4**), one new resveratrol derivative (**5**) with a dihydrobenzofuran skeleton, together with two known natural stilbene trimers (**6** and **7**), and six known natural stilbene dimers (**8**–**13**) were isolated and structurally identified (Figure 1). Among these substances, trimers **6** and **7** were obtained for the first time through direct transformation from resveratrol. Moreover, their potential formation mechanisms were also discussed.

## 2. Results and Discussion

### 2.1. Synthesis of ***2*** with Resveratrol as a Starting Material and HRP and Hydrogen Peroxide-Catalyzed Biotransformation of ***1*** and ***2***

**Figure 2:**
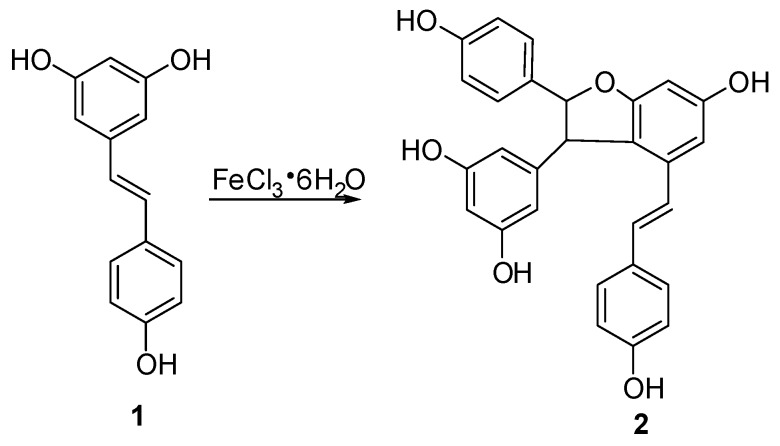
Semi-synthetic route of compound **2**.

In this study, semisynthetic (±)-ε-viniferin (**2**) under the procedure shown in Figure 2 was used as the starting material for biotransformation. According to the method reported in our previous paper [17], *trans*-resveratrol was subjected to an oxidative coupling reaction in aqueous methanol using FeCl_3_·6H_2_O as an oxidant, and this procedure was performed in combination with column chromatography on silica gel. Thus, **2** with 13.5% yield was produced.

Successively, biotransformation of **1** and **2** in aqueous acetone catalyzed by horseradish peroxidase and hydrogen peroxide generated a major product peak **8** and a complicated mixture (Figure S30 in Supplementary Materials), which resulted in the isolation and identification of four resveratrol trimers (**3**, **4**, **6**, and **7**, where **3** and **4** are new ones), one new resveratrol derivative **5**, and six known dimers (**8**–**13**) (Figure 1). Their structures and stereochemistry were elucidated by analyzing spectroscopic data.

### 2.2. Structure Elucidation of Biotransformation Products

**Figure 3:**
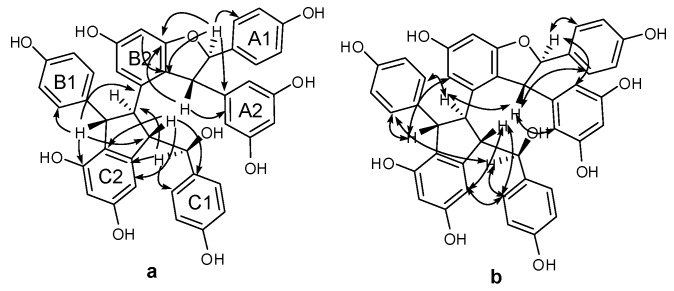
Important HMBC (**a**) and NOESY (**b**) interactions of **3**.

Compound **3** was obtained as a brown amorphous powder. The negative ion peak at *m*/*z* 697.2076 [M − H]^−^ (cacld. for C_42_H_33_O_10_, 697.2079) in its HR-ESI-MS (Figure S7) corresponded to the molecular formula of C_42_H_34_O_10_ and indicated that **3** could be a resveratrol trimer. The IR spectrum (Figure S9) revealed the presence of hydroxyls (ν_max_ = 3395 and 3187 cm^−1^) and aromatic rings (ν_max_ = 1600, 1513, and 1469 cm^−1^). Absorption bands were also observed at λ_max_ (log ε) 203 (5.14), 231 (4.66), and 283 (3.92) nm in the UV spectrum (Figure S8). The ^1^H-NMR spectrum (Figure S1, Table 1) showed three sets of A_2_B_2_ systems for rings A1, B1, and C1 at δ_H_ of 6.83 (2H, d, *J* = 8.4 Hz, H-3(5)a), 7.24 (2H, d, *J* = 8.4 Hz, H-2(6)a), 6.54 (2H, d, *J* = 8.4 Hz, H-3(5)b), 6.34 (2H, d, *J* = 8.4 Hz, H-2(6)b); 6.68 (2H, d, *J* = 8.4 Hz, H-3(5)c), and 6.85 (2H, d, *J* = 8.4 Hz, H-2(6)c); one set of AB_2_ system for ring A2 at δ_H_ of 6.36 (1H, t, *J* = 2.4 Hz, H-12a) and 6.20 (2H, d, *J* = 2.4 Hz, H-10(14)a); two sets of *meta*-coupled aromatic protons for rings B2 and C2 at δ_H_ of 5.98 (1H, d, *J* = 2.4 Hz, H-14b) and 6.19 (1H, d, *J* = 2.4 Hz, H-12b), and δ_H_ 5.17 (1H, d, *J* = 1.8 Hz, H-14c) and 6.24 (1H, d, *J* = 1.8 Hz, H-12c). The ^1^H-NMR spectrum also displayed the presence of two mutually coupled benzyl methine protons at δ_H_ of 5.33 (1H, d, *J* = 3.0 Hz, H-7a) and 4.83 (1H, d, *J* = 3.0 Hz, H-8a) and a sequence of successively coupled benzyl methine protons at δ_H_ of 4.30 (1H, brs, H-7b), 3.76 (1H, brs, H-8b), 4.02 (1H, dd, *J* = 4.2, 3.0 Hz, H-7c), and 2.96 (1H, m, H-8c). Moreover, the HSQC spectrum (Figure S4) supplied the complete assignment of all protonated carbon, as shown in Table 1. The ^13^C-NMR spectrum (Figure S2, Table 1) of **3** revealed the presence of six aliphatic carbons at δ_C_ of 52.64, 54.45, 65.07, 77.10, 54.73, and 93.78 ppm and 36 aromatic carbons. The aliphatic carbon at δ_C_ of 77.10 ppm was due to an alcohol carbon. The carbon signals at δ_C_ of 54.73 and 93.78 ppm and proton signals at δ_H_ of 5.33 and 4.83 suggested the presence of dihydroxybenzofuran ring. The three remaining aliphatic protons and three carbon atoms indicated the presence of an indane ring. This group of evidence demonstrated that **3** possessed a skeleton similar to that of davidiol B [18,19]. In addition, compared with those of davidiol B, the downfield shifts of H-2(6)a, H-7a, H-8a, H-10(14)a, H-8b, and H-2(6)c in **3** (∆δ_H_ +0.24, +0.28, +1.72, +0.29, +0.95, and +0.50 ppm) caused by the anisotropic effect of the aromatic ring suggested that **3** could be a 7c-empimer of davidiol B [20]. In the HMBC spectrum (Figure S5 and Figure 3a) of **3**, the correlations of H-2c, H-6c, H-14c, and C-7c, which were attached to the hydroxyl group, showed that C-7c was excluded from the additional ring, and ring C1 was attached at C-7c. The correlations between H-7b, H-8b, H-5b, and C-1b verified that ring B1 was connected at C-7b. Moreover, the correlations between H-7a and C-10(14)a and H-8a and C-2(6)a substantiated that ring A1 was linked at C-7a, and ring A2 was linked at C-8a. Comparison of the spectral data with those of davidiol B and the analysis of DEPT, HMBC, and HSQC (Figures S3~S5) correlations determined the planar structure of **3** (Figure 1).

The stereochemistry of **3** was determined by analyzing the NOESY spectrum (Figure S6 and Figure 3b). The interactions among H-2(6)b and H-7b, H-8b suggested a *trans* orientation of rings B1 and B2. The NOE interactions among H-7c and H-2(6)c, H-2(6)b suggested a *cis* orientation of rings C1 and B1. The NOE interactions between H-8a and H-2(6)a and between H-7a and H-10(14)a indicated a *trans* orientation between rings A1 and A2. Furthermore, the NOE interactions among H-14c and H-2(6)c, H-8c revealed that ring C1 could be located near ring C2. The downfield shift of H-14c [6.68–6.65 (m, 1H)] and upfield shift of H-8b [2.81 (s, 1H)] in **3** compared with those of davidiol B further substantiated this observation. Therefore, the structure of **3** was determined as shown in Figure 1.

**Figure 4:**
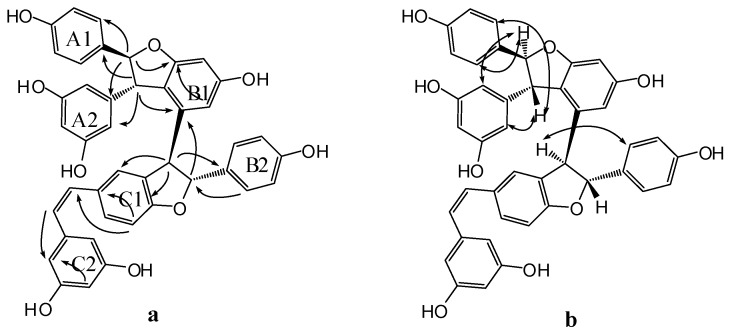
Significant HMBC (**a**) and NOESY (**b**) correlations of compound **4**.

Compound **4** was obtained as a light brown amorphous powder. Its corresponding negative HR-ESI-MS (Figure S4) *m*/*z* 679.1973 [M − H]^−^ (cacld. for C_42_H_31_O_9_, 679.1968) showed a molecular formula of C_42_H_31_O_9_, which implied that **4** could be a resveratrol trimer. The UV (λ_max_ = 203.4 (4.99), 228 (4.69), and 281 (4.22) nm) and IR (ν_max_ = 3335, 1604, 1516, 1487, 1449, 1004, and 836 cm^−1^) spectra (Figures S18 and S19) of **4** displayed the presence of a phenolic oligostilbene containing a *cis* olefinic bond [21]. The ^1^H- and ^13^C-NMR spectral data (Figures S10 and S11, Table 1) of **4**, along with ^1^H-^1^H COSY, DEPT, HSQC, and HMBC spectra (Figures S12~S14 and S16), revealed resonances attributable to two 4-hydroxyphenyl groups (rings A1 and B1), two 3,5-dihydroxyphenyl groups (rings A2 and C2), a 3,5-dihydroxy-1,2-disubstituted benzene ring (ring B2), and a 4-hydroxy-1,3-disubstituted benzene ring (ring C1). The ^1^H-NMR spectral data also suggested the presence of two sets of aliphatic signals at δ_H_ of 5.22 (1H, d, *J* = 6.1 Hz, H-7a) and 3.85 (1H, d, *J* = 6.1 Hz, H-8a). These signals are characteristic of a 2,3-diaryldihydrobenzofuran moiety, except a *cis*-1,2-disubstituted olefinic bond at δ_H_ 6.21 (1H, d, *J* = 12.0 Hz, H-7c) and 6.05 (1H, d, *J* = 12.0 Hz, H-8c). Our comparison revealed that the NMR spectroscopic data of **4** were remarkably similar to those of *cis*-diptoindonesin B reported in the literature [21], which suggested that **4** possessed the same planar structure as *cis*-diptoindonesin B. In the HMBC spectrum (Figure S14 and Figure 4a) of **4**, the significant correlations of H-7a/C-2(6)a, C-9a, and C-11b; H-8a/C-10(14)a, C-1a, and C-9b; H-7b/C-2(6)b, and C-9b; and H-8b/C-1b, C-2c, and C-4c; H-7c/C-5c; H-8c/C-2(6)c, in combination with their shifts, further supported the planar structure of **4** as shown in Figure 1.

The relative stereochemistry of H-7a/H-8a and H-7b/H-8b was determined by analyzing the NOESY spectrum of **4** (Figure S15 and Figure 4b). The NOE interactions among H-7a and H-2(6)a, H-10(14)a and those among H-8a and H-2(6)a, H-10(14a) suggested a *trans* orientation between H-7a and H-8a. Similarly, the NOE interactions between H-8b and H-2(6)b also suggested a *trans* relationship of H-7b and H-8b. Despite that **4** exhibited a planar structure similar to that of *cis*-diptoindonesin B, their difference indicated that **4** could be a stereoisomer of *cis*-diptoindonesin B. The comparison of the NMR data of **4** with *cis*-diptoindonesin B demonstrated that H-8a, H-8c, and H-10(14)c and C-8b, C-9b, C-8c, and C-9c in **4** were shifted by ∆δ_H_ of +0.36, −0.24, −0.19 and ∆δ_C_ of +3.3, +4.7, −2.5, and −3.5 ppm, respectively. This evidence demonstrated that the relative configurations of C-7b and C-8b in **4** could be 7b*R* and 8b*R* instead of 7b*S* and 8b*S* in *cis*-diptoindonesin B, respectively [21]. In contrast to the relative configuration of *rel*-(7a*S*, 8a*S*, 7b*S*, and 8b*S*) in *cis*-diptoindonesin B, the relative configurations of **4** for C-7a, C-8a, C-7b, and C-8b were determined as *rel*-(7a*S*, 8a*S*, 7b*R*, and 8b*R*), respectively (Figure 1). This conjecture was confirmed by the fact that H-8a and H-2(6)b were deshielded because of anisotropic effects induced by rings B2 and C1. On the contrary, H-7c, H-8c, and H-10(14)c were shielded due to anisotropic effects elicited by rings B2.

**Figure 5:**
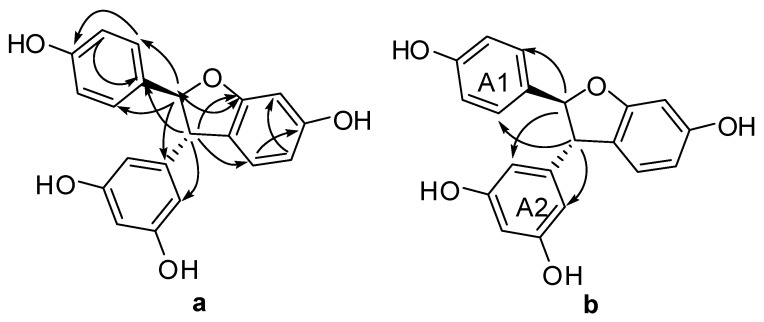
Significant HMBC (**a**) and NOESY (**b**) correlations of compound **5**.

Compound **5** was obtained as brown amorphous powder. The corresponding positive ion HR-ESI-MS (Figure S27) peak at *m*/*z* 337.1073 [M + H]^+^ (cacld. for C_20_H_17_O_5_, 337.1071) provided the molecular formula of C_20_H_16_O_5_. The IR spectrum (Figure S29) displayed the presence of hydroxyls (3337 cm^−1^) and aromatic rings (1615, 1517, and 1464 cm^−1^). The ^1^H-NMR spectrum (Figure S20, Table 1) showed one A2B2 system at δ_H_ of 7.20 (2H, d, *J* = 8.5 Hz, H-3(5)a) and 6.82 (2H, d, *J* = 8.5 Hz, H-3(5)a), one AB2 system at δ_H_ of 6.25 (1H, t, *J* = 2.1 Hz, H-12a) and 6.16 (2H, d, *J* = 2.1 Hz, H-10(14)a), one ABX system at δ_H_ of 6.47 (1H, d, *J* = 2.2 Hz, H-3b) and 6.67 (2H, brs, H-5b, H-6b), and two mutually coupled benzyl methine protons at δ_H_ of 5.34 (1H, d, *J* = 8.3 Hz, H-7a) and 4.36 (1H, d, *J* = 8.3 Hz, H-7a). In addition, the carbon signals at δ_C_ of 54.66 and 92.53 ppm in ^13^C-NMR (Figure S21, Table 1), together with the proton signals at δ_H_ of 5.34 and 4.36 ppm, suggested the presence of dihydroxybenzofuran ring. Accordingly, **5** was identified as a resveratrol derivative with benzofuran skeleton, as shown in Figure 1, which was confirmed by DEPT, HSQC, HMBC, COSY, and UV spectra (Figures S22~S26 and S28). In HMBC spectrum (Figure S24 and Figure 5a), the correlations between H-7a and C-2(6)a, C-9a, C-2b and H-8a and C-10(14)a, C-1a, C-6b suggested that ring A1 could be linked at C-7a, and ring A2 could be linked at C-8a. In NOESY experiment (Figure S25 and Figure 5b), the NOE enhancements between H-7a and H-2(6)a/H-10(14)a and H-8a and H-10(14)a/H-2(6)a suggested a *trans* orientation of rings A1 and A2. Therefore, the structure of **5** was determined as shown in Figure 1.

In addition to these three compounds, two known resveratrol trimers, namely, davidiol B (**6**) [18] and *rel*-(7a*S*,8a*S*,7b*S*,8b*S*)-*cis*-diptoindonesin B (**7**) [21], and six known dimers, namely, (±)-resveratrol-*trans*-dehydrodimer (**8**) [22,23], (±)-resveratrol-*cis*-dehydrodimer (**9**) [23,24], leachianol G (**10**) [25,26], leachianol F (**11**) [25,26], parthenostilbenin B (**12**) [20], and ampelopsin B (**13**) [27,28], were identified by comparing their physical and spectroscopic data with those reported in previous studies. Among them, trimers **6** and **7**, which are difficult to obtain by common organic reactions, were obtained for the first time by direct biotransformation from resveratrol.

### 2.3 Probable Coupling Reaction Mechanisms

**Figure 6:**
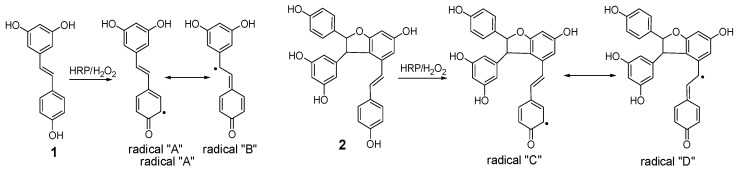
Plausible radical intermediates from **1** and **2** by horseradish peroxidase and hydrogen peroxide.

**Figure 7:**
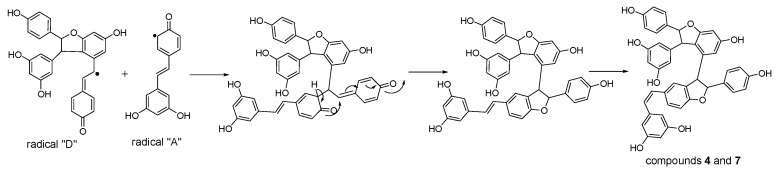
Proposed formation mechanisms for compounds **4** and **7**.

**Figure 8:**
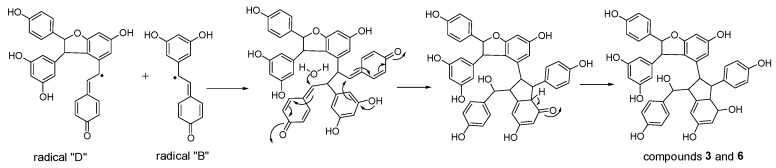
Proposed formation mechanisms for compounds **3** and **6**.

Considering the obtained results, a mechanism on how different trimeric derivatives formed were proposed. HRP-catalyzed biotransformation is presumed on the basis of radical reaction [13,14,29]. Induced by hydrogen peroxide, resveratrol, and (±)-ε-viniferin were dehydrogenated and rearranged to form different radicals (Figure 6). Afterward, these HRP-catalyzed radicals were combined to produce different dimers and trimers. The coupling of one radical D and one radical A, and subsequent tautomeric rearrangement and intramolecular nucleophilic attack to the intermediate quinone yielded the dihydrofuran trimers **4** and **7** (Figure 7). Consequently, the formation of **3** and **6** can be easily explained by the coupling of one radical D with one radical B and subsequent addition of a water molecule to the intermediate quinone (Figure 8). Furthermore, **5** may be formed through the oxidation of (±)-ε-viniferin by H_2_O_2_ and HRP, but an appropriate account for the reactivity cannot be established with this evidence. In addition, the formation mechanisms of the dimers obtained in this work are the same as those reported in our previous paper [26,30,31].

## 3. Materials and Methods

### 3.1. Materials and Instrumentation

Optical rotations were measured on P2000 polarimeter (JASCO, Tokyo, Japan). UV spectra were obtained on a JASCOP650 spectrometer (JASCO). IR spectra were recorded on a Nicolet 5700 FT-IR microscope instrument (FT-IR microscope transmission, Thermo Electron Corporation, Madison, WI, USA). 1D and 2D NMR spectra were acquired at 500 or 600 MHz for ^1^H and 125 or 150 MHz for ^13^C, respectively, on Varian INOVA 500 MHz, or Bruker AVANCE III HD 600 MHz (Bruker Corporation, Karlsruhe, Germany), in acetone-*d*_6_ or methanol-*d*_4_, with solvent peaks as references. ESI-MS and HR-ESI-MS data were measured using an AccuToFCS JMST100CS spectrometer (Agilent Technologies, Ltd., Santa Clara, CA, USA). Column chromatography (CC) was performed with silica gel (200–300 mesh, Qingdao Marine Chemical Inc., Qingdao, China). HPLC separation was performed on an instrument consisting of a Waters 515 pump and a Waters 2487 dual λ absorbance detector (Waters Corporation, Milford, MA, USA) with a YMC semi-preparative column (250 × 10 mm i.d.) packed with C18 (5 μM). TLC was carried out with glass precoated silica gel GF254 plates (Qingdao Marine Chemical, Inc., Qingdao, China). Spots were visualized under UV light or by spraying with 7% H_2_SO_4_ in 95% EtOH followed by heating.

### 3.2. Synthesis of Compoud ***2***

The solution of FeCl_3_·6H_2_O (380 g, 1.43 mol) in H_2_O (100 mL) was added to a solution of **1** (300 g, 1.32 mol) in methanol (500 mL) under stirring at room temperature, and the mixture was stirred for 60 h at room temperature. After removing of methanol in vacuo, water was added to the mixture, and the mixture was extracted with EtOAc. Subsequently, the obtained organic layer was washed with brine and water, dried over anhydrous Na_2_SO_4_ for 24 h, then concentrated in vacuo to give a residue, which was further chromatographed on silica gel column with CHCl_3_–MeOH (15:1, *v*/*v*) as eluent to provide unreacted resveratrol 130 g and product **2** (23 g, yield 13.5%).

*Compound* **2:** grey amorphous powder. ^1^H-NMR(CD_3_COCD_3_, 500 MHz) δ: 8.41 (OH), 8.38 (OH), 8.33 (OH), 8.17 (2H, 2 × OH), 7.20 (2H, d, *J* = 9.0 Hz, H-2a, 6a), 7.16 (2H, d, *J* = 8.5 H, H-2b, 6b), 6.90 (1H, d, *J* = 16.0 Hz, H-7b), 6.82 (2H, d, *J* = 9.0 Hz, H-3a, 5a), 6.73 (2H, d, *J* = 8.5 Hz, H-3b, 5b), 6.72 (2H, d, *J* = 2.0 Hz, H-10a, 14a), 6.70 (1H, d, *J* = 16.0 Hz, H-8b), 6.32 (1H, d, *J* = 2.0 Hz, H-12b), 6.23 (2H, br s, H-12a, 14b), 5.41 (1H, d, *J* = 5.5 Hz, H-7a), 4.47 (1H, d, *J* = 5.5 Hz, H-8a); (+)-ESI *m*/*z*: 477 [M + Na]^+^.

### 3.3. Treatment of ***1*** and ***2*** with Horseradish Peroxidase/Hydrogen Peroxide

To a mixed solution of **1** (3000 mg, 13.2 mmol) and **2** (1500 mg, 3.3 mmol) in acetone (240 mL), 50 mL of water was added under stirring at room temperature. After that, a solution of HRP (10.0 mg) in water (30 mL) was added slowly. The reactant was stirred for 5 min, 30% H_2_O_2_ (1.4 mL) was then added. The reactant was stirred for another 7 h at room temperature. Finally, the reaction mixture was suspended in water, and extracted with ethyl acetate. The organic layer was dried on anhydrous Na_2_SO_4_ for 24 h, and concentrated in vacuo to yield a residue.

The residue was subjected to column chromatography over ODS, eluting with a gradient of increasing methanol in water (15~100%) to provide nine fractions (R-I~R-IX) on the basis of HPLC analysis. R-VI (2.5 g) was further fractionated via silica gel column chromatography (CC), eluting with CHCl_3_–MeOH (17:1, *v*/*v*) to yield **8** (0.493 g, 16.5%) and R-VI-2~R-VI-4. R-VI-3 was then separated by semi-preparative Rp-HPLC (column, Rp-18, 250 × 10 mm, 5 µm) eluted using methanol/water (56:44, *v*/*v*) to afford **9** (2 mg, 0.07%); 30 mg R-I (total 280 mg) was separated by semi-preparative Rp-HPLC using a mobile phase of methanol/water (16:84, *v*/*v*) to afford **10** (9 mg, 2.7%) and **11** (4 mg, 1.2%). R-II (30 mg) was further purified by semi-preparative Rp-HPLC eluted with a mobile phase of MeOH–H_2_O (30:70, *v*/*v*) to afford **5** (1.7 mg). 300 mg R-III (total amount 600 mg) was resolved by Rp-MPLC with a gradient of increasing MeOH (18~30%) in water to give R-III-1~R-III-3. Among them, Fraction R-III-2 (34 mg) were subsequently separated by Rp-HPLC with a mobile phase of methanol/water (40:60, *v*/*v*) to afford **6** (5 mg, 0.43%) and **3** (3 mg, 0.26%); R-III-1 and R-III-3 were dealt with the same manner to provide **13** (13 mg, 0.87%), and **12** (7 mg, 0.44%), respectively. Furthermore, R-VII (600 mg) was subjected to Rp-MPLC eluting with methanol–water (49:51, *v*/*v*) to yield R-VII-1 and R-VII-2; subsequently, R-VII-1 was purified by semi-preparative Rp-HPLC using a mobile phase of methanol–water (46:54, *v*/*v*) to provide **7** (2 mg); R-VII-2 was dealt with methanol–water (54:46, *v*/*v*) by semi-preparative Rp-HPLC in the same manner to give **4** (6 mg).

*Compound* **3**: Brown amorphous powder. UV (MeOH) λ_max_ (log ε): 203 (5.14), 231.2 (4.66), 283 (3.92) nm; IR (film) ν_max_: 3395, 3187, 2921, 2850, 1646, 1513, 1469, 1420, 1342, 1245, 1120, 1005, 834, 722, 648 cm^−1^; ^1^H-NMR (600 MHz, MeOD) and ^13^C-NMR (150 MHz, MeOD), see Table 1; (+)-ESI *m*/*z*: 721.0 [M + Na]^+^, 736.9 [M + K]^+^; (−)-ESI *m*/*z*: 696.9 [M − H]^−^, 732.9 [M + Cl]^−^; HR-ESI-MS *m*/*z*: 697.2076, [M − H]^−^ (cacld. for C_42_H_33_O_10_, 697.2079).

*Compound* **4**: Light brown amorphous powder. UV (MeOH) λ_max_ (log ε): 203.4 (4.99), 228 (4.69), 281 (4.22) nm; IR (film) ν_max_: 3335, 2924, 2852, 1604, 1516, 1487, 1449, 1349, 1241, 1169, 1004, 836, 694 cm^−1^; ^1^H-NMR (600 MHz, MeOD) and ^13^C-NMR (150 MHz, MeOD), see Table 1; (+)-ESI *m*/*z*: 703.3 [M + Na]^+^; (−)-ESI *m*/*z*: 678.9 [M − H]^−^, 715.8 [M + Cl]^−^; HR-ESI-MS *m*/*z*: 679.1973, [M − H]^−^ (cacld. for C_42_H_31_O_9_, 679.1968).

*Compound* **5**: Brown amorphous powder. UV (MeOH) λ_max_ (log ε): 203.4 (4.73), 229.8 (4.29), 283 (3.70) nm; IR (film) ν_max_: 3337, 2921, 2852, 1615, 1517, 1488, 1464, 1351, 1235, 1198, 1157, 1105, 1000, 836, 778, 746, 691 cm^−1^; ^1^H-NMR (600 MHz, acetone-*d*_6_) and ^13^C-NMR (150 MHz, acetone-*d*_6_), see Table 1; HR-ESI-MS *m*/*z*: 337.1073 [M + H]^+^ (cacld. for C_20_H_17_O_5_, 337.1071).

*Davidiol B* (**6**): Brown amorphous powder. ^1^H-NMR (600 MHz, acetone-*d*_6_), δ: 7.00 (2H, d, *J* = 8.5 Hz, H-2(6)a), 6.87 (2H, d, *J* = 8.5 Hz, H-3(5)a), 6.68–6.65 (1H, m, H-14c), 6.53 (2H, d, *J* = 8.6 Hz, H-3(5)b), 6.45 (2H, d, *J* = 8.5 Hz, H-3(5)c), 6.38 (1H, d, *J* = 2.1 Hz, H-12c), 6.35 (2H, d, *J* = 8.4 Hz, H-2(6)c), 6.31 (1H, t, *J* = 2.2 Hz, H-12a), 6.16 (1H, d, *J* = 2.1 Hz, H-12b), 6.13 (2H, d, *J* = 8.4 Hz, H-2(6)b), 5.99 (1H, d, *J* = 2.1 Hz, H-14b), 5.91 (2H, d, *J* = 2.0 Hz, H-10(14)a), 5.05 (1H, d, *J* = 2.3 Hz, H-7a), 4.10 (1H, s, H-7b), 3.95 (1H, d, *J* = 10.1 Hz, H-7c), 3.11 (1H, d, *J* = 2.4 Hz, H-8a), 2.90 (1H, d, *J* = 10.1 Hz, H-8c), 2.81 (1H, s, H-8b). ^13^C-NMR (150 MHz, acetone-*d*_6_), δ: 161.74s (C-11b), 159.94s (C-11(13)a), 159.83s (C-13b), 158.71s (C-13c), 157.90s (C-4a), 157.48s (C-4c), 155.84s (C-4b), 154.98s (11c), 150.15s (C-9c), 148.81s (C-9a), 148.01s (C-9b), 136.55s (C-1b), 136.18s (C-1c), 134.35s (C-1a), 129.72d (C-2(6)b), 128.66d (C-2(6)c), 127.57d (C-2(6)a), 122.69s (C-10c), 118.50s (C-10b), 116.41d (C-3(5)c), 116.02d (C-3(5)a), 115.18d (C-3(5)b), 107.41d (C-14c), 106.90d (C-10(14)a, 104.52d (C-14b), 102.11d (C-12c), 101.70d (C-12a), 63.97d (C-8c), 54.57d (C-8b), 54.46d (C-8a), 53.24d (C-7b). (+)-ESI *m*/*z*: 721.0 [M + Na]^+^, 736.9 [M + K]^+^; (−)-ESI *m*/*z*: 696.9 [M − H]^−^, 732.9 [M + Cl]^−^.

*cis-Diptoindonesin B* (**7**): Brown amorphous powder. ^1^H-NMR (600 MHz, methanol-*d*_4_), δ: 7.09 (1H, d, *J* = 8.6 Hz, H-6c), 6.99 (2H, d, *J* = 8.6 Hz, H-2(6)b), 6.92 (2H, d, *J* = 8.5 Hz, H-2(6)a), 6.75 (2H, d, *J* = 8.6 Hz, H-3(5)a), 6.68 (2H, d, *J* = 8.5 Hz, H-3(5)b), 6.63 (1H, brs, H-2c), 6.60 (1H, d, *J* = 8.3 Hz, H-5c), 6.37 (1H, d, *J* = 12.2 Hz, H-7c), 6.31 (1H, d, *J* = 12.2 Hz, H-8c), 6.25 (1H, d, *J* = 2.1 Hz, H-12b), 6.22 (2H, d, *J* = 2.2 Hz, H-10(14)c), 6.19 (1H, d, *J* = 2.1 Hz, H-14b), 6.12 (1H, t, *J* = 2.2 Hz, H-12c), 6.10 (1H, t, *J* = 2.2 Hz, H-12a), 5.85 (2H, d, *J* = 2.2 Hz, H-10(14)a), 5.15 (1H, d, *J* = 10.0 Hz, H-7b), 5.14 (1H, d, *J* = 5.2 Hz, H-7a), 4.29 (1H, d, *J* = 10.0 Hz, H-8b), 3.49 (1H, d, *J* = 5.2 Hz, H-8a). ^13^C-NMR (150 MHz, methanol-*d*_4_), δ: 162.61s (C-11b), 160.55s (C-4c), 160.25s (C-13b), 159.86s (C-11(13)a), 159.12s (C-11(13)c), 158.81s (C-4b), 158.41s (C-4a), 147.58s (C-9a), 140.93s (C-9c), 140.29s (C-9b), 133.87s (C-1a), 131.92s (C-1b), 131.73s (C-1c), 131.12s (C-3c), 130.32d (C-6c), 129.53d (C-8c), 128.94d (C-2(6)b), 128.17d (C-2(6)a), 127.23d (C-2c), 122.03s (C-10b), 116.36d (C-3(5)b), 116.28d (C-3(5)a), 109.68d (C-5c), 108.53d (C-10(14)c), 108.13d (C-14b), 107.09d (C-10(14)a), 102.56d (C-12c), 102.18d (C-12a), 96.83d (C-12b), 94.99d (C-7b), 94.55d (C-7a), 56.84d (C-8a), 55.52d (C-8b). (+)-ESI-MS *m*/*z*: 681.0 [M + H]^+^, 703.0 [M + Na]^+^; (−)-ESI *m*/*z*: 678.9 [M − H]^−^, 714.9 [M + Cl]^−^.

*Ampelopsin B* (**13**): Brown amorphous powder. ^1^H-NMR (500 MHz, methanol-*d*_4_), δ: 7.02 (2H, d, *J* = 8.6 Hz, H-2(6)b), 6.89 (2H, d, *J* = 7.9 Hz, H-2(6)a), 6.69 (2H, d, *J* = 8.6 Hz, H-3(5)b), 6.60 (2H, d, *J* = 8.6 Hz, H-3(5)a), 6.30 (1H, d, *J* = 2.2 Hz, H-12b), 6.27 (1H, d, *J* = 1.9 Hz, H-14a), 6.09 (1H, d, *J* = 1.8 Hz, H-14b), 6.01 (1H, d, *J* = 2.0 Hz, H-12a), 5.65 (1H, d, *J* = 11.4 Hz, H-7b), 5.14 (1H, t, *J* = 3.8 Hz, H-7a), 4.05 (1H, d, *J* = 11.4 Hz, H-8b), 3.53 (1H, dd, *J* = 17.4, 4.1 Hz, H-8aα), 3.17 (1H, brd, *J* = 17.4 Hz, H-8aβ). (+)-ESI *m*/*z*: 455.0 [M + H]^+^, 477.0 [M + Na]^+^; (−)-ESI *m*/*z*: 453.0 [M − H]^−^, 489.0 [M + Cl]^−^.

## 4. Conclusions

The HRP-catalyzed biotransformation of **1** and **2** produced various resveratrol stilbene oligomers, including dimers, trimers, and tetramers. In this reaction mixture, four resveratrol trimers (**3**, **4**, **6**, and **7**), one new resveratrol derivative (**5**) with a dihydrofuran skeleton, and six dimers (**8**–**13**) were isolated and identified. Among these compounds, **3** and **4** were newly identified in our study. The raceme nature of the dimers was indicated by the zero values of their optical rotations, and this finding suggested that a radical mechanism was involved in HRP-catalyzed biotransformation. Our study favored the enzymatic biotransformation of stilbenes by HRP as a prominent method to produce oligomeric stilbenes for research activity. Considering that these new compounds may occur naturally as minor constituents, we observed that our reference data provided a basis for the detection of the presence of these stilbene oligomers in future investigations. Oligostilbenes were reported to show various activities [1,2]. Therefore, these products should be further examined, and results will be reported in our future research.

## Figures and Tables

**Table 1:** ^1^H- and ^13^C-NMR spectroscopic data of compounds **3**–**5** *.

No.	3		4		5	
1a	134.69s		133.85s		131.98s	
2(6)a	128.25d	7.24 (d, 8.4)	128.80d	7.00 (d, 9.0)	127.59d	7.20 (d, 8.5)
3(5)a	116.04d	6.83 (d, 8.4)	116.36d	6.80 (d, 9.0)	115.14d	6.82 (d, 8.5)
4a	158.11s		158.58s		157.36s	
7a	93.78d	5.33 (d, 3.0)	95.00d	5.22 (d, 6.1)	92.53d	5.34 (d, 8.3)
8a	54.73d	4.83 (d, 3.0)	57.8d	3.85 (d, 6.1).	57.66d	4.36 (d, 8.3)
9a	149.28s		147.28s		144.29s	
10a	107.13d	6.20 (d, 2.4)	107.27d	5.94 (d, 2.4)	106.51d	6.16 (d, 2.1)
11a	159.93s		159.56s		158.79s	
12a	101.82d	6.36 (t, 2.4)	101.90d	6.09 (t, 2.4)	101.34d	6.25 (t, 2.1)
13a	159.93s		159.56s		158.79	
14a	107.13d	6.20 (d, 2.4)	107.27d	5.94 (d,2.4)	106.51d	6.16 (d, 2.1)
1b	137.41s		131.87s		153.02s	
2b	129.35d	6.34 (d, 8.4)	128.53d	7.14 (d, 8.4)	131.30s	
3b	115.48d	6.54 (d, 8.4)	116.30d	6.75 (d, 8.4)	112.10d	6.47 (d, 2.2)
4b	155.85s		158.69s		151.57s	
5b	115.48d	6.54 (d, 8.4)	116.30d	6.75 (d, 8.4)	109.08d	6.67 (brs)
6b	129.35d	6.34 (d, 8.4)	128.53d	7.14 (d, 8.4)	114.69d	6.67 (brs)
7b	52.64d	4.30 (s, 1H)	94.86dd	5.30 (d, 8.4)		
8b	54.45d	3.76 (s, 1H)	58.78d	4.30 (d, 8.4)		
9b	147.43s		145.04s			
10b	119.71s		120.40s			
11b	161.56s		162.74s			
12b	95.45d	6.19 (d, 2.4)	96.83d	6.23 (d, 2.4)		
13b	159.93s		159.48s			
14b	104.82d	5.98 (d, 2.4)	109.40d	6.25 (d, 2.4)		
1c	136.42s		131.51s			
2c	129.22d	6.85 (d, 8.4)	127.36d	6.76 (d, 2.4)		
3c	115.33d	6.68(d, 8.4)	132.74s			
4c	157.34s		160.43s			
5c	115.33d	6.68 (d, 8.4)	109.85d	6.65 (d, 8.4)		
6c	129.22d	6.85 (d, 8.4)	130.09d	6.98 (dd, 8.4, 2.4)		
7c	77.10d	4.02 (dd, 4.2, 3.0)	131.70d	6.21 (d, 12.0)		
8c	65.07d	2.96 (m)	127.04d	6.05 (d, 12.0)		
9c	148.34s		137.54s			
10c	123.66s		107.71d	6.03 (d, 2.4)		
11c	154.66s		159.79s			
12c	102.22d	6.24 (d, 1.8)	102.54d	6.17 (t, 2.4)		
13c	158.38s		159.79s			
14c	106.66d	5.17 (d, 1.8)	107.71d	6.03 (d, 2.4)		

* Data (δ_H_) were measured in MeOD for ^1^H-NMR at 600 MHz and for ^13^C-NMR at 150 MHz. The assignments were based on DEPT, ^1^H-^1^H COSY, HSQC, HMBC, and NOESY experiments, respectively.

## Supplementary Materials

The following are available online. Figures S1~S9: ^1^H-NMR, ^13^C-NMR, DEPT, HSQC, HMBC, NOESY, HRESIMS, UV, and IR spectra in CD_3_COCD_3_ of **3**. Figures S10~S19: ^1^H-NMR, ^13^C-NMR, DEPT, HSQC, HMBC, NOESY, COSY, HRESIMS, UV, and IR spectra in CD_3_COCD_3_ of **4**. Figures S20~S29: ^1^H-NMR, ^13^C-NMR, DEPT, HSQC, HMBC, NOESY, COSY, HRESIMS, UV, and IR spectra in CD_3_COCD_3_ of **5**. Figure S30: HPLC chromatogram of biotransformation products of **1** and **2**.
