# Supplementary material for: Biomimetic Synthesis of Resveratrol Trimers Catalyzed by Horseradish Peroxidase
Source: Molecules. 2017 May 17;22(5):819. doi: 10.3390/molecules22050819 (PMC6154677; doi:10.3390/molecules22050819)
Supplement: Supplementary file 1 [file molecules-22-00819-s001.pdf]

Supplementary data

Biomimetic Synthesis of Resveratrol Trimers Catalyzed by Horseradish Peroxidase

Jian-Qiao Zhang [1], Gan-Peng Li [2], Yu-Long Kang [1], Bin-Hao Teng [1] and Chun-Suo Yao[1,*]

CONTENTS

S1. ^{1}H-NMR spectrum of compound **3** in CD_3COCD_3.

S2. ^{13}C-NMR spectrum of compound **3** in CD_3COCD_3.

S3. DEPT spectrum of compound **3** in CD_3COCD_3.

S4. HSQC spectrum of compound **3** in CD_3COCD_3.

S5. HMBC spectrum of compound **3** in CD_3COCD_3.

S6. NOESY spectrum of compound **3** in CD_3COCD_3.

S7. HRESIMS spectrum of compound **3**.

S8. UV spectrum of compound **3** in CH_3OH.

S9. IR spectrum of compound **3**.

S10. ^{1}H-NMR spectrum of compound **4** in CD_3OD.

S11. ^{13}C-NMR spectrum of compound **4** in CD_3OD.

S12. DEPT spectrum of compound **4** in CD_3OD.

S13. HSQC spectrum of compound **4** in CD_3OD.

S14. HMBC spectrum of compound **4** in CD_3OD.

S15. NOESY spectrum of compound **4** in CD_3OD.

S16. COSY spectrum of compound **4** in CD_3OD.

S17. HRESIMS spectrum of compound **4**.

S18. UV spectrum of compound **4** in CH_3OH.

S19. IR spectrum of compound **4**.

S20. ^{1}H-NMR spectrum of compound **5** in CD_3COCD_3.

S21. ^{13}C-NMR spectrum of compound **5** in CD_3COCD_3。

S22. DEPT spectrum of compound **5** in CD_3COCD_3.

S23. HSQC spectrum of compound **5** in CD_3COCD_3.

S24. HMBC spectrum of compound **5** in CD_3COCD_3.

S25. NOESY spectrum of compound **5** in CD_3COCD_3.

S26. COSY spectrum of compound **5** in CD_3COCD_3.

S27. HRESIMS spectrum of compound **5**.

S28. UV spectrum of compound **5** in CH_3OH.

S29. IR spectrum of compound **5**.

S30. HPLC chromatogram of biotransformation products of **1** and **2**.

S1. ^{1}H-NMR spectrum of compound **3** in CD_3COCD_3.

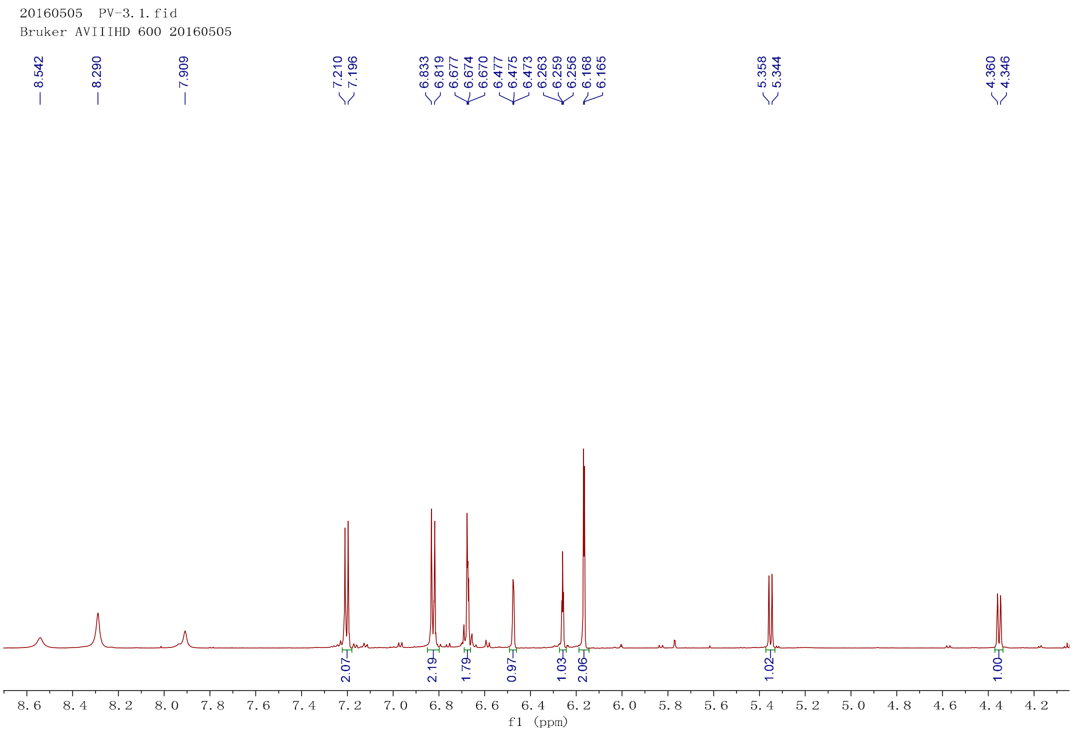

S2. ^{13}C-NMR spectrum of compound **3** in CD_3COCD_3.

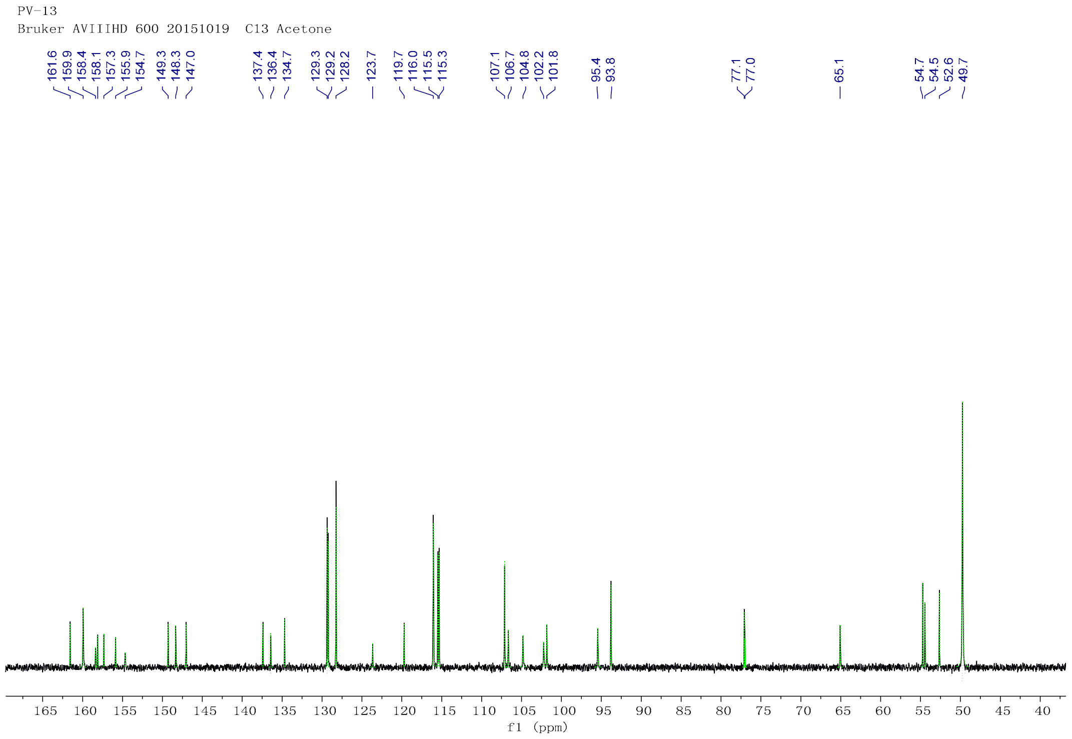

S3. DEPT spectrum of compound **3** in CD_3COCD_3.

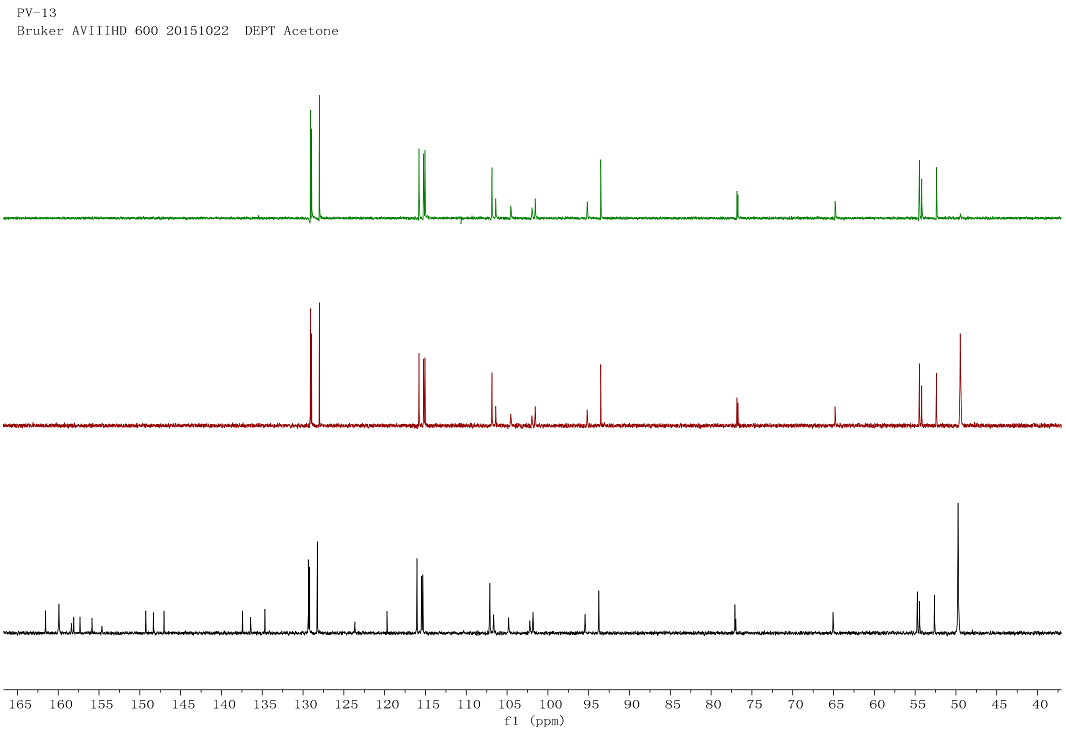

S4. HSQC spectrum of compound **3** in CD_3COCD_3.

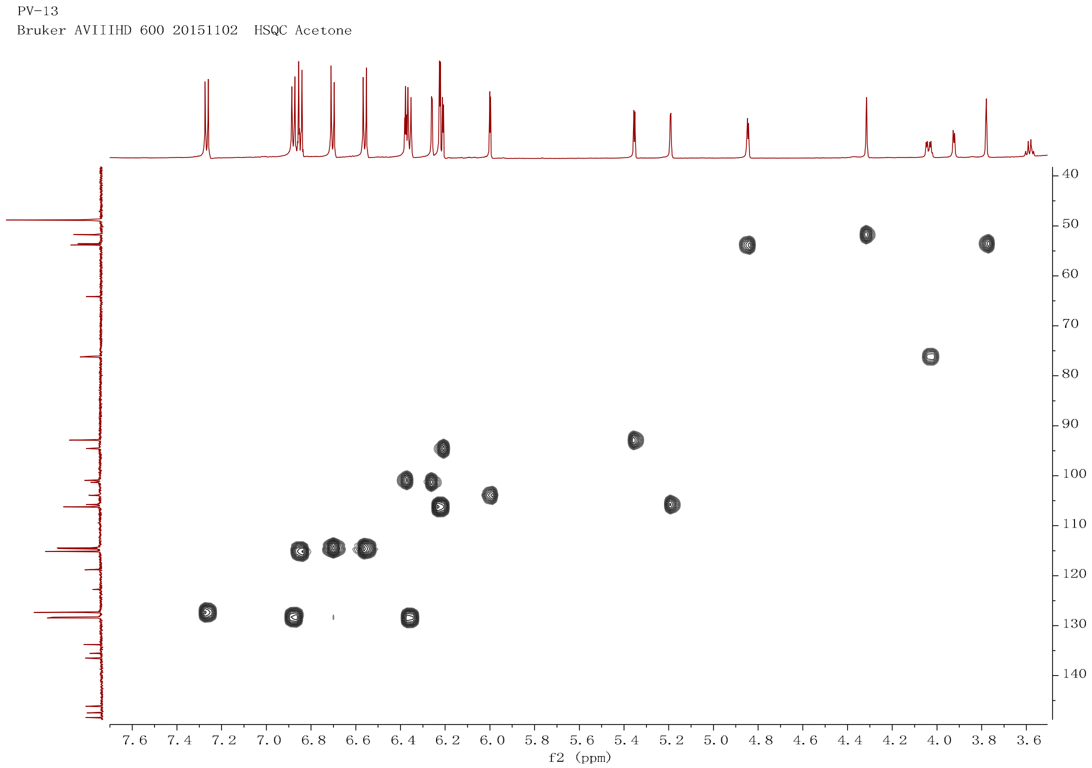

S5. HMBC spectrum of compound **3** in CD_3COCD_3.

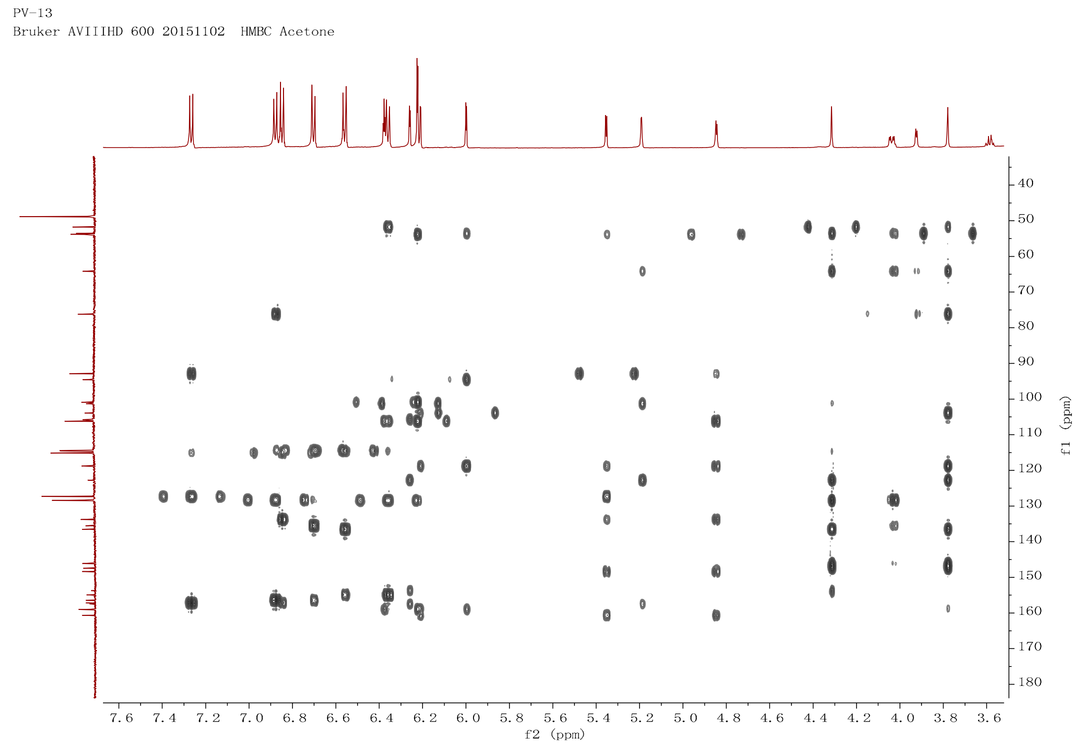

S6. NOESY spectrum of compound **3** in CD_3COCD_3.

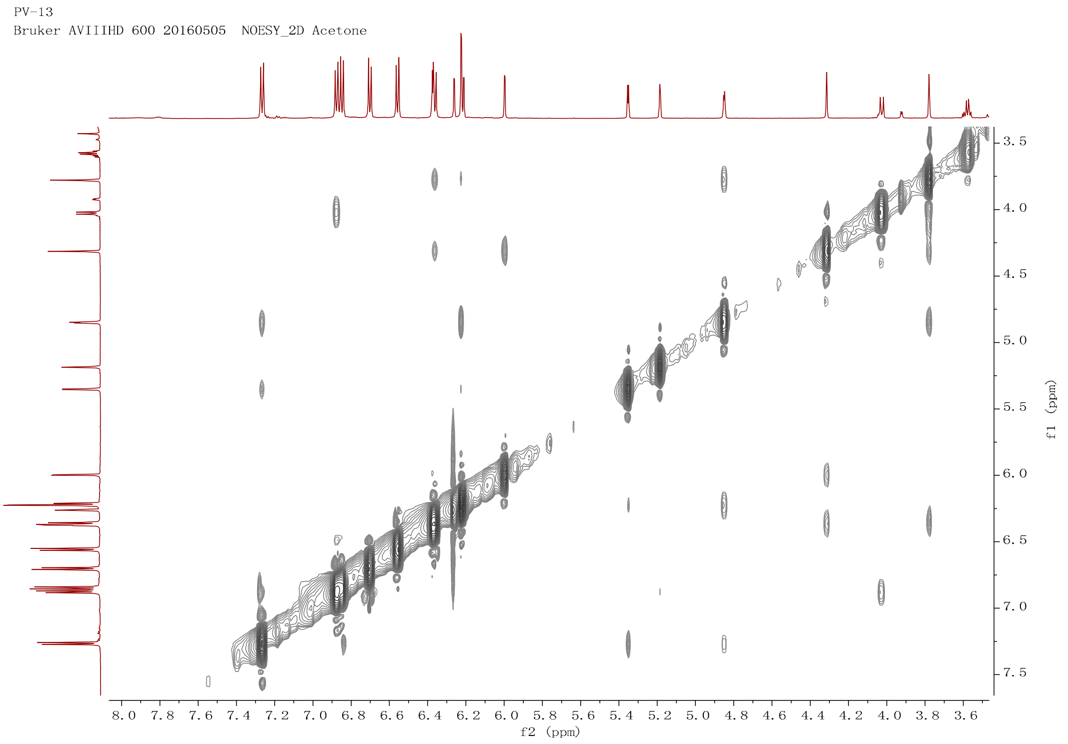

S7. HRESIMS spectrum of compound **3**.

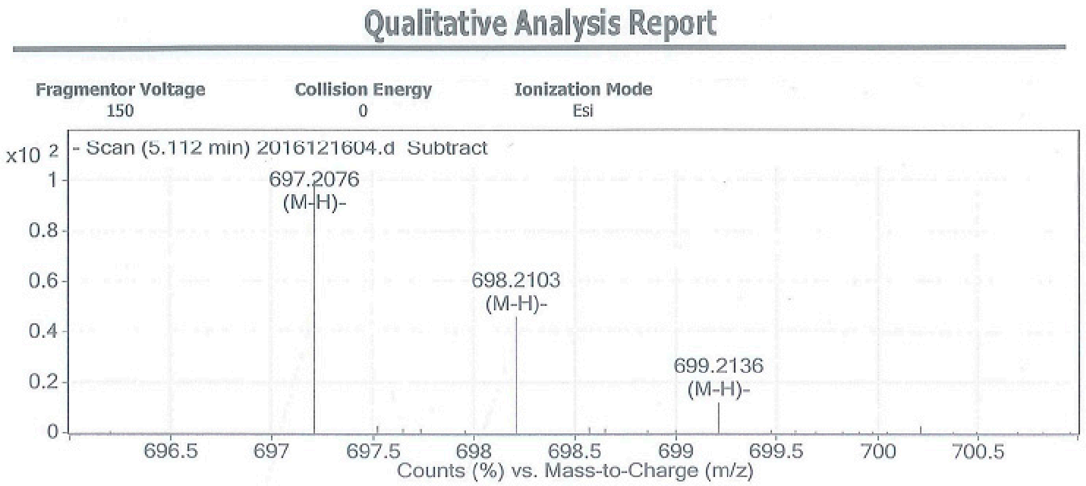

m/z	Ion	Formula	Abundance
697.2076	(M-H)	[illegible]	[illegible]

Best	Formula (M)	Ion Formula	Score	Cross Sco	Mass	Calc Mass	Calc m/z	Diff (ppm)	Abs Diff (ppm)	Mass Match	Abund Match	Spacing Match	DBE
✓	[illegible]	[illegible]	[illegible]		[illegible]	[illegible]	[illegible]	0.61	0.61	[illegible]	[illegible]	[illegible]	[illegible]
	C37 H34 N2 O13	C37 H33 N2 O13	[illegible]		[illegible]	[illegible]	[illegible]	[illegible]	[illegible]	[illegible]	[illegible]	[illegible]	[illegible]
	[illegible]	[illegible]	[illegible]		[illegible]	[illegible]	[illegible]	[illegible]	[illegible]	[illegible]	[illegible]	[illegible]	20
	[illegible]	[illegible]	[illegible]		[illegible]	[illegible]	[illegible]	[illegible]	[illegible]	[illegible]	[illegible]	[illegible]	21
	[illegible]	[illegible]	[illegible]		[illegible]	[illegible]	[illegible]	[illegible]	[illegible]	100	[illegible]	99.7	17
	C30 H38 N2 O17	C30 H37 N2 O17	98.03		698.2148	698.217	697.2068	[illegible]	[illegible]	[illegible]	[illegible]	[illegible]	13
	[illegible]	[illegible]	[illegible]		[illegible]	[illegible]	697.2068	1.82	1.82	[illegible]	[illegible]	[illegible]	[illegible]
	C39 H38 N2 O7 S2	C39 H37 N2 O7 S2	97.7		[illegible]	[illegible]	[illegible]	[illegible]	[illegible]	[illegible]	[illegible]	99.43	21
	[illegible]	[illegible]	[illegible]		[illegible]	[illegible]	697.2106	4.46	4.46	[illegible]	[illegible]	[illegible]	12
	C47 H38 S3	C47 H37 S3	[illegible]		[illegible]	[illegible]	[illegible]	-1.77	1.77	[illegible]	[illegible]	[illegible]	[illegible]
	[illegible]	[illegible]	[illegible]		[illegible]	[illegible]	[illegible]	[illegible]	[illegible]	[illegible]	[illegible]	[illegible]	16
	[illegible]	[illegible]	[illegible]		[illegible]	[illegible]	697.2044	-4.42	4.42	[illegible]	[illegible]	[illegible]	4
	[illegible]	[illegible]	[illegible]		[illegible]	[illegible]	[illegible]	[illegible]	[illegible]	[illegible]	[illegible]	99.14	3

S8. UV spectrum of compound **3** in CH_3OH.

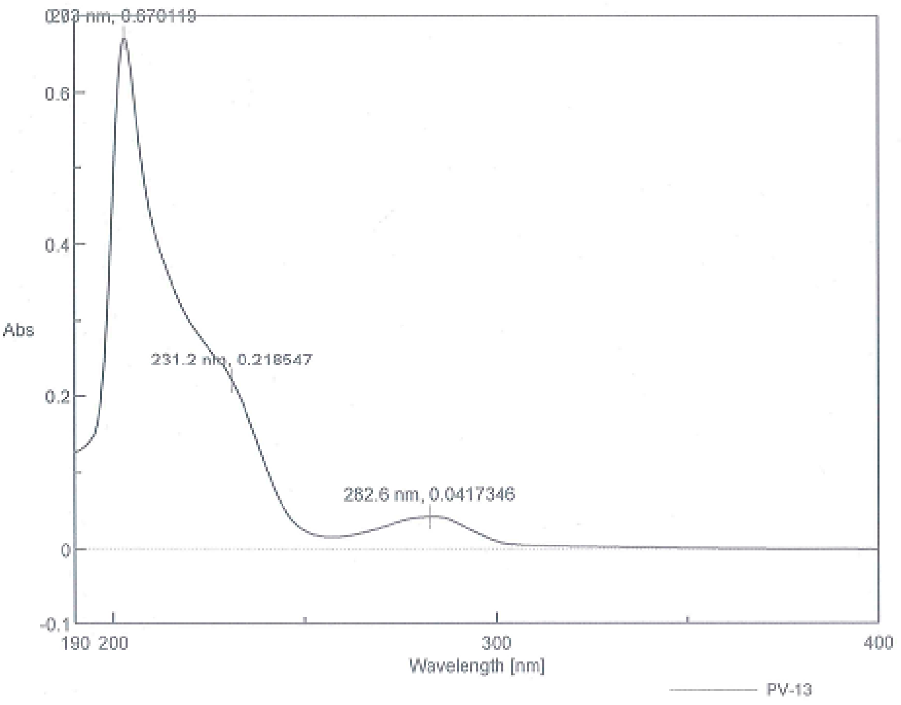

S9. IR spectrum of compound **3**.

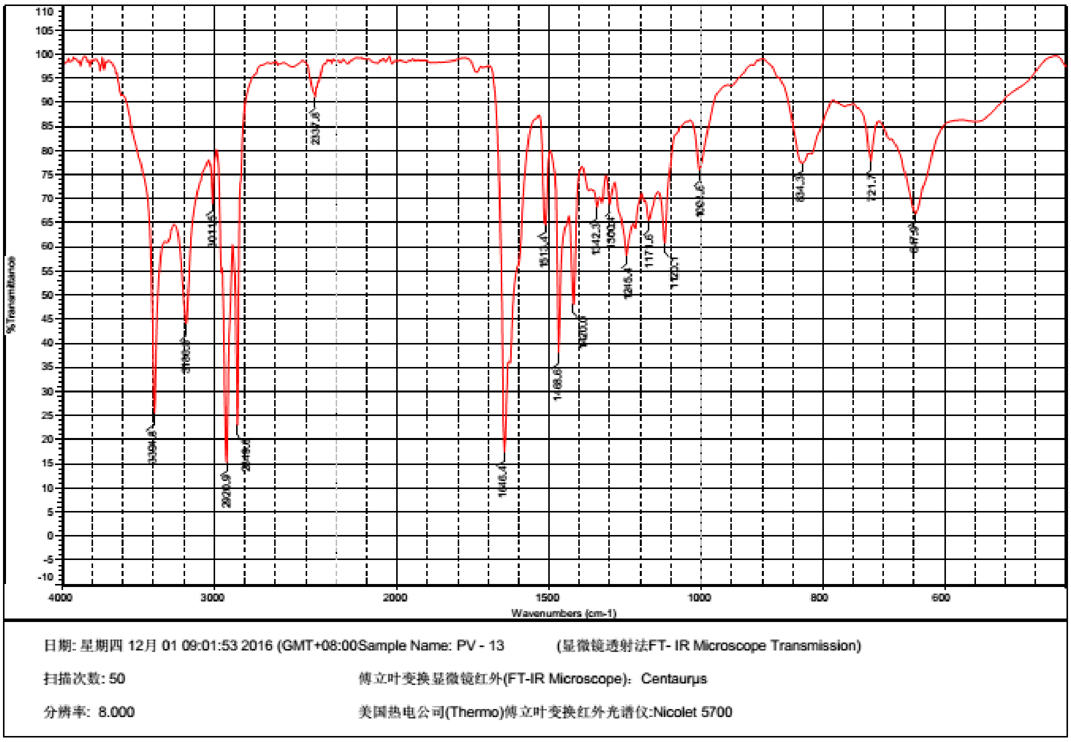

S10. ^{1}H-NMR spectrum of compound **4** in CD_3OD.

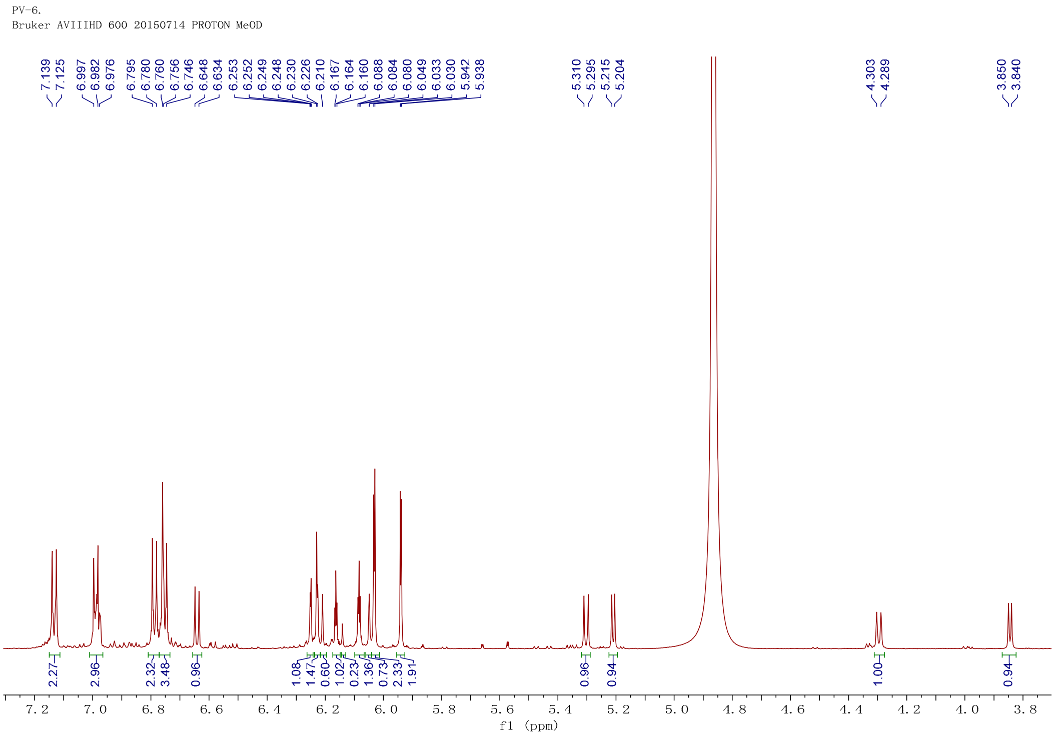

S11.^{13}C-NMR spectrum of compound **4** in CD_3OD.

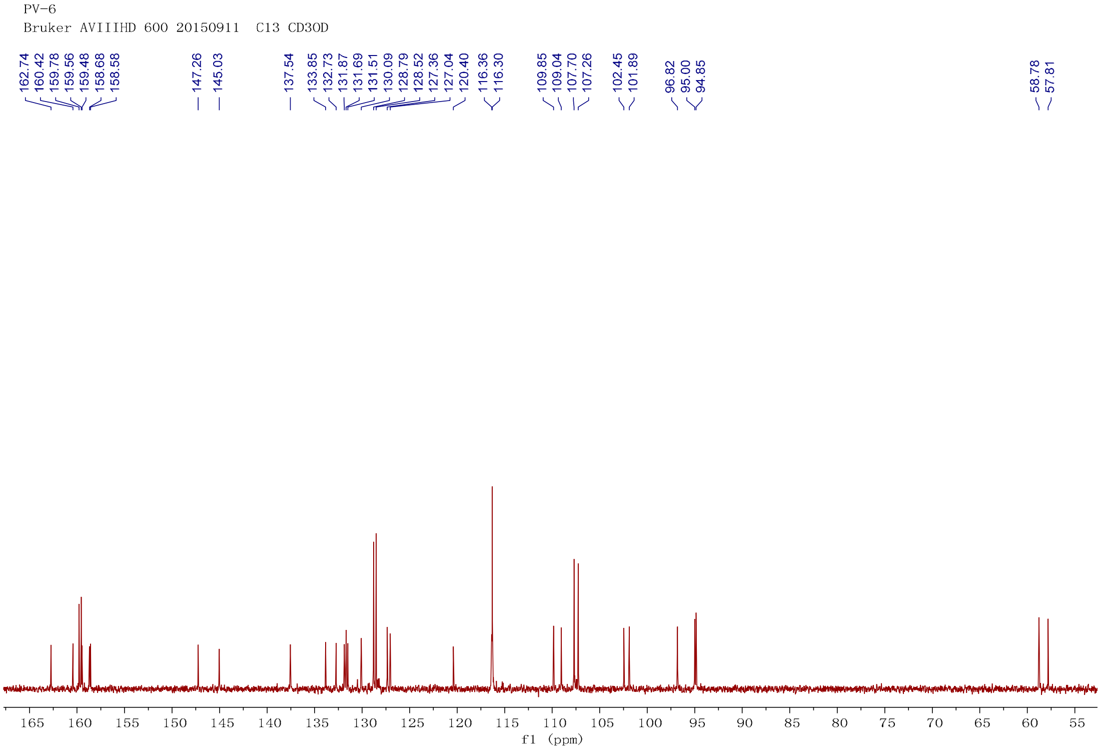

S12. DEPT spectrum of compound **4** in CD_3OD.

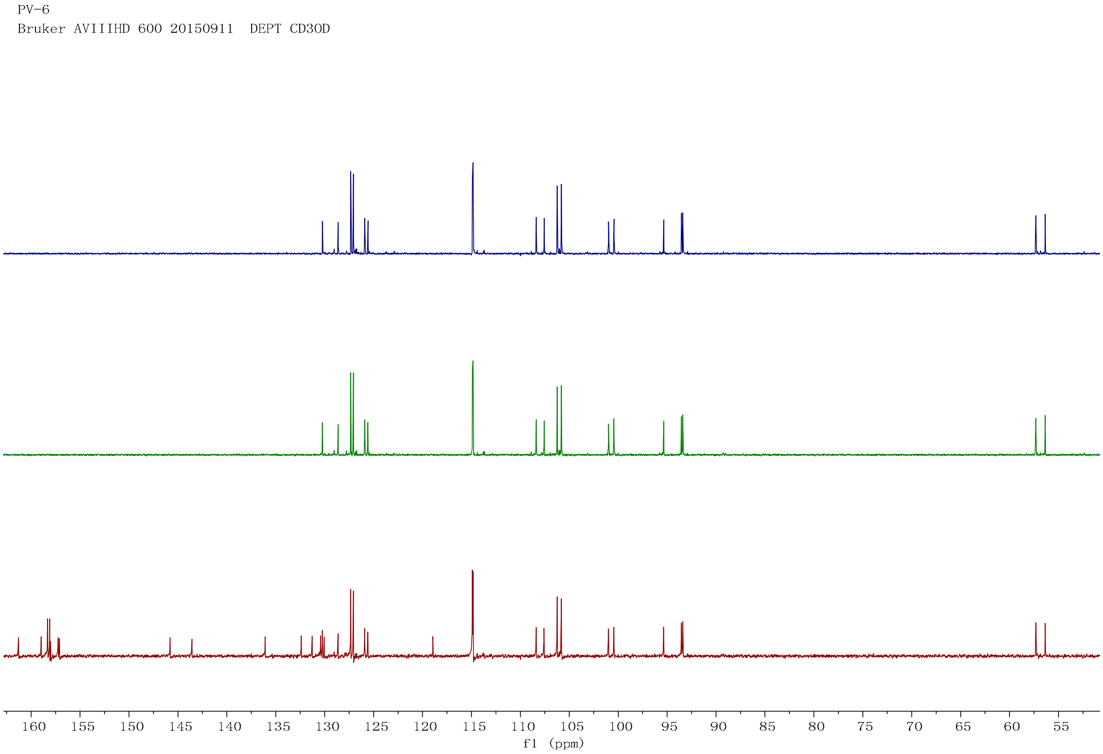

S13. HSQC spectrum of compound **4** in CD_3OD.

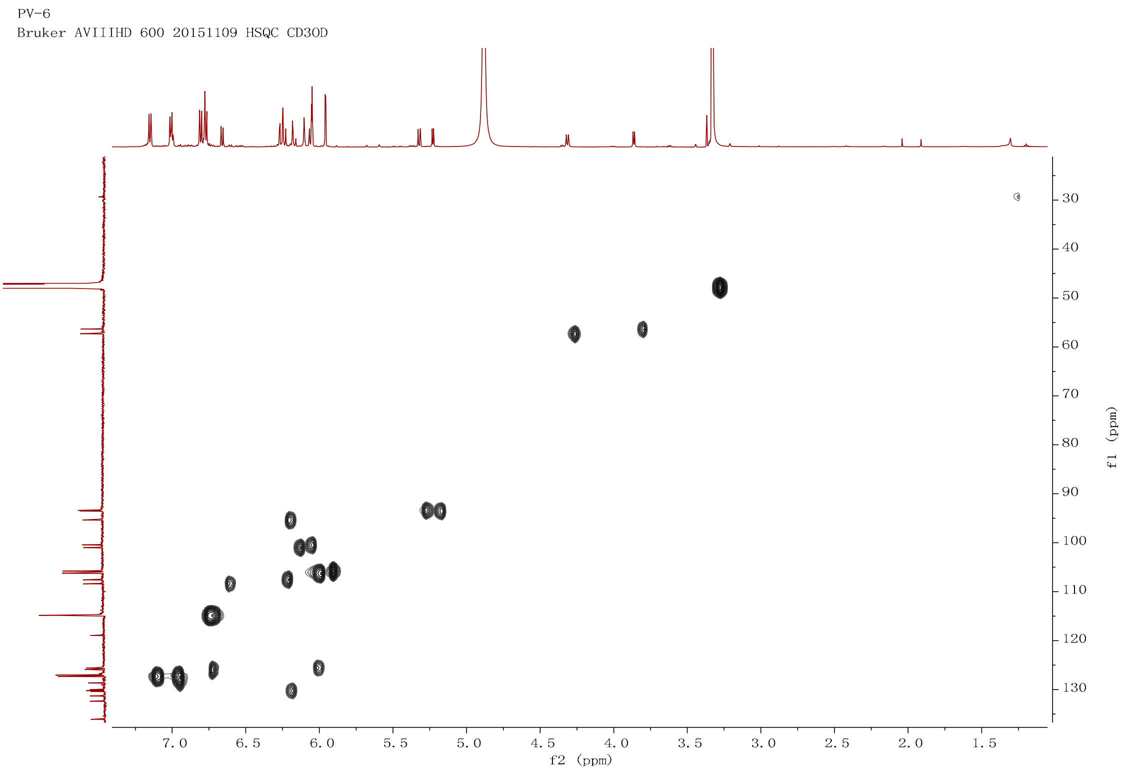

S14. HMBC spectrum of compound **4** in CD_3OD.

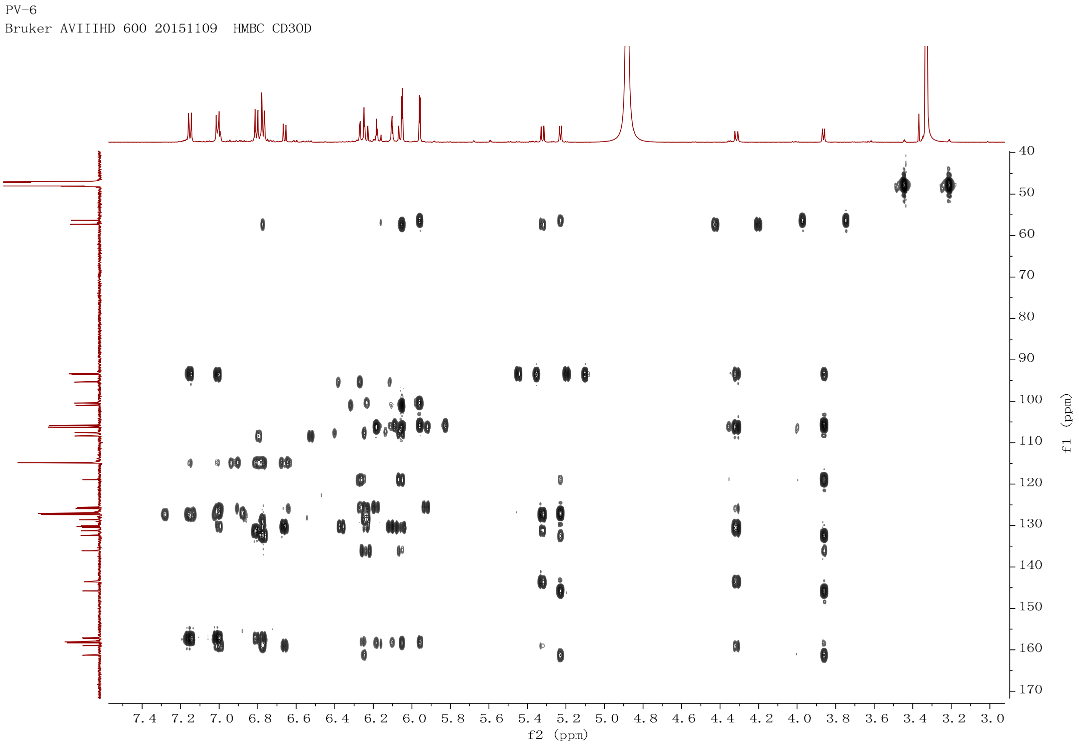

S15. NOESY spectrum of compound **4** in CD_3OD.

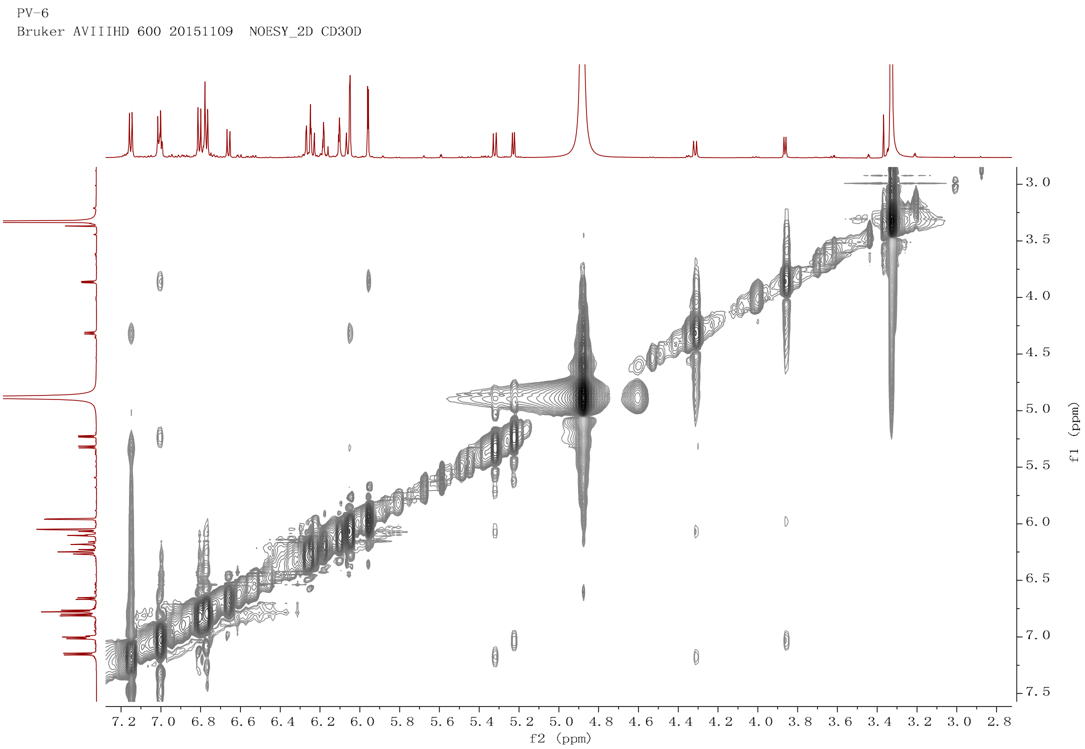

S16. COSY spectrum of compound **4** in CD_3OD

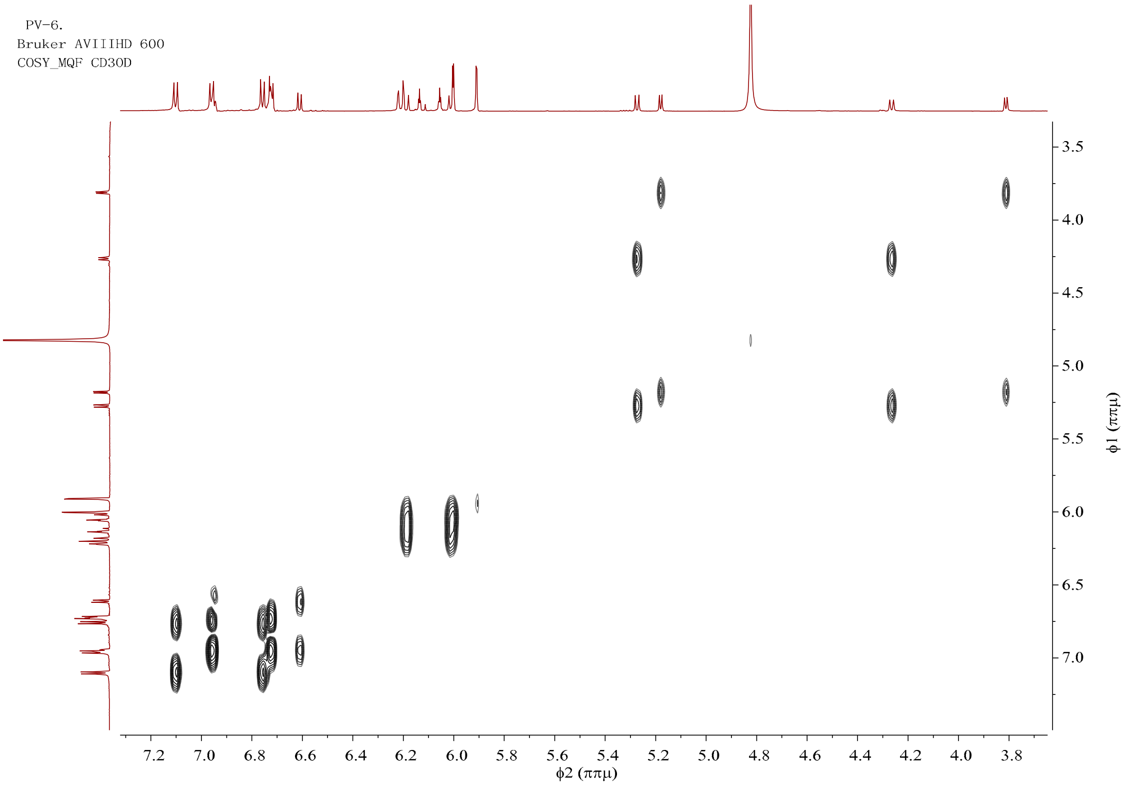

S17. HRESIMS spectrum of compound **4**.

Qualitative Analysis Report

Fragmentor Voltage	Collision Energy	Ionization Mode
150	0	Esi

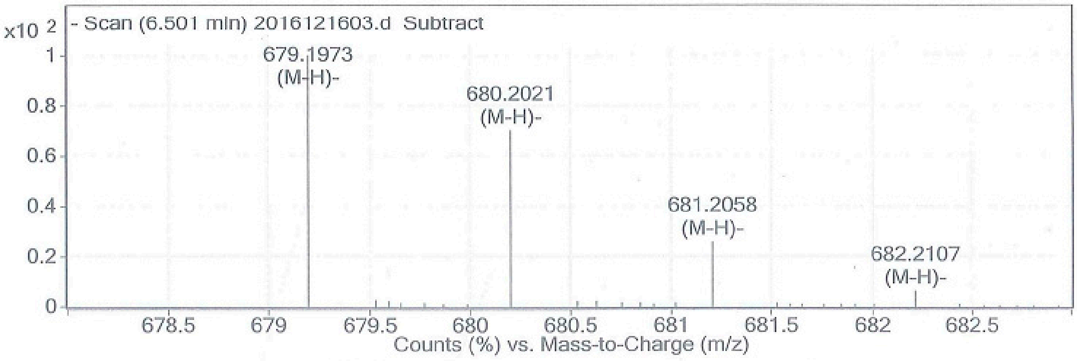

m/z	Ion	Formula	Abundance
679.1973	(M-H)-	C42 H31 O9	74522.8

Best	Formula (M)	Ion Formula	Score	Cross Sco	Mass	Calc Mass	Calc m/z	Diff (ppm)	Abs Diff (ppm)	Mass Match	Abund Match	Spacing Match	DBE
☑	C42 H32 O9	C42 H31 O9	96.85		680.2046	680.2046	679.1974	0.46	0.46	99.99	96.72	99.74	27
☐	C46 H32 O1 S	C46 H31 O1 S	97.28		680.205	680.2021	679.1949	-4.21	4.21	99.37	92.16	99.24	31
☐	C43 H36 O4 S2	C43 H35 O4 S2	96.73		680.205	680.2055	679.1982	0.69	0.69	99.96	87.97	98.04	23
☐	C39 H36 O5 S	C39 H35 O9 S	94.32		680.205	680.203	679.2007	4.41	4.41	99.31	83.8	98.08	22
☐	C38 H36 N2 O6 S2	C38 H35 N2 O6 S2	91.2		680.2051	680.2015	679.1943	-5.28	5.28	99.01	82.59	98.41	22
☐	C40 H40 O4 S3	C40 H39 O4 S3	93.87		680.2051	680.2088	679.2016	5.59	5.59	98.89	81.28	98.09	21
☐	C34 H36 N2 O11 S	C34 H35 N2 O11 S	93.06		680.205	680.204	679.1967	-1.56	1.56	99.91	76.79	98.88	18
☐	C35 H40 N2 O6 S3	C35 H39 N2 O6 S3	92.51		680.2051	680.2048	679.1975	-0.38	0.38	99.99	75.59	97.83	17

S18. UV spectrum of compound **4** in CH_3OH.

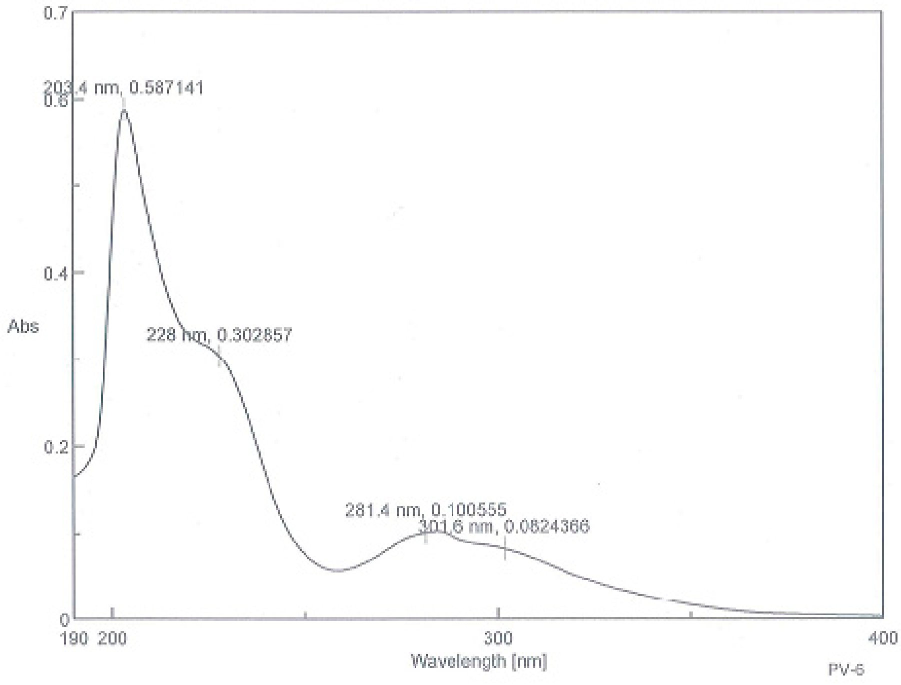

S19. IR spectrum of compound **4**.

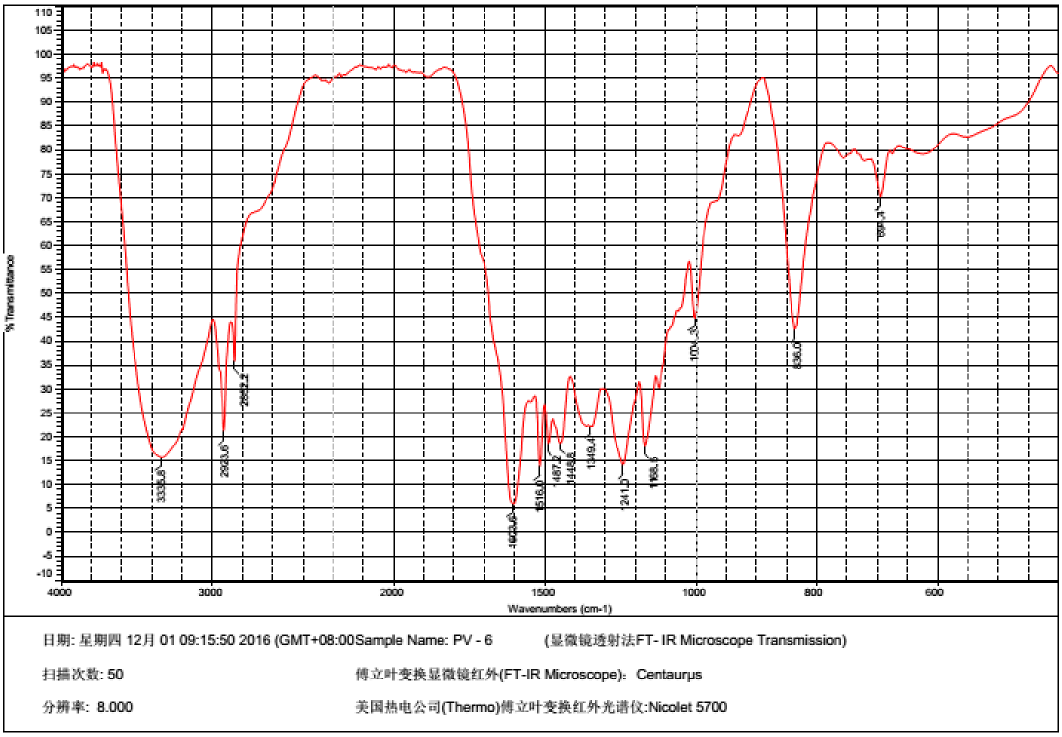

S20. ^{1}H-NMR spectrum of compound **5** in CD_3COCD_3.

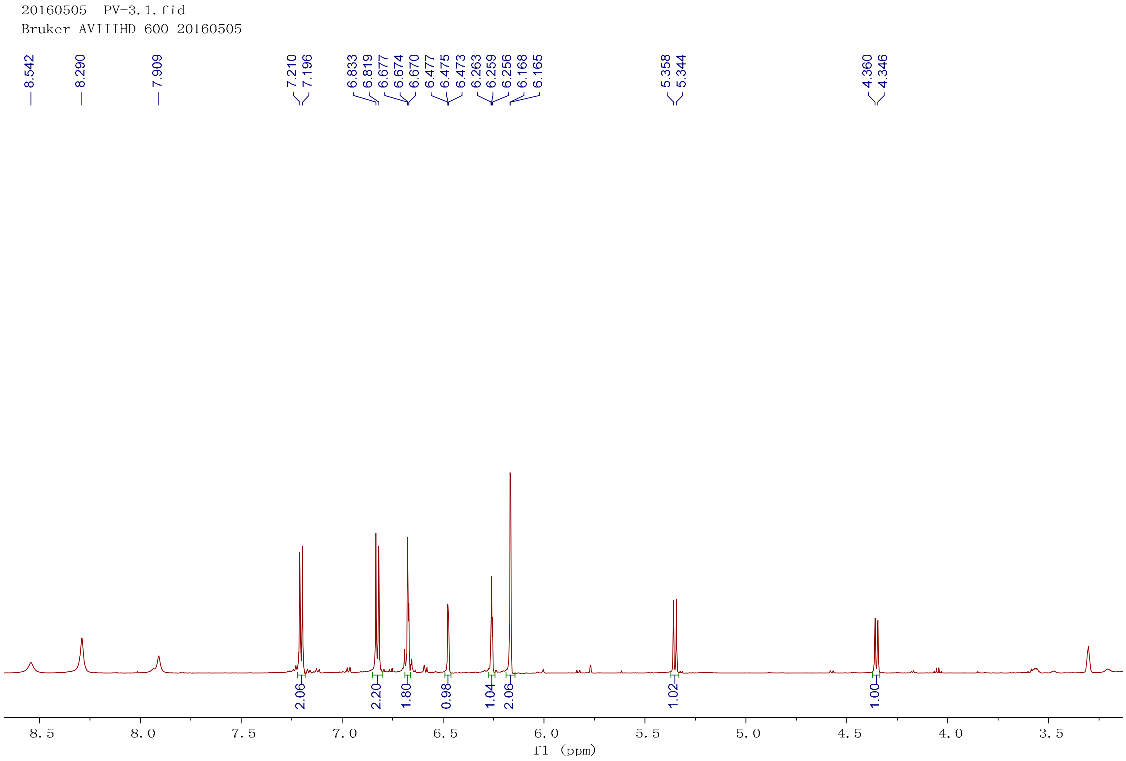

S21. ^{13}C-NMR spectrum of compound **5** in CD_3COCD_3。

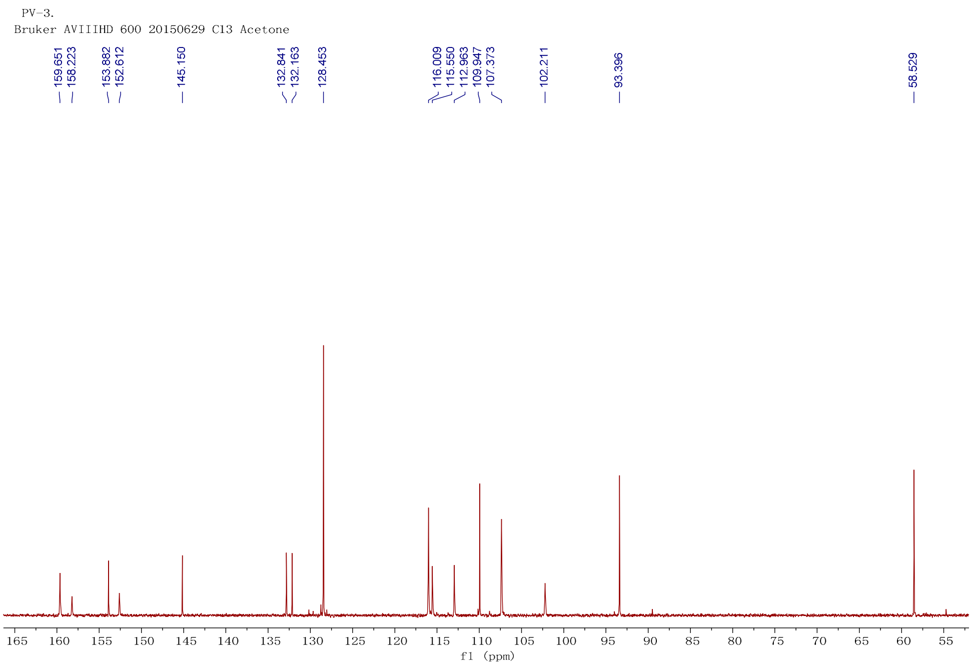

S22. DEPT spectrum of compound **5** in CD_3COCD_3.

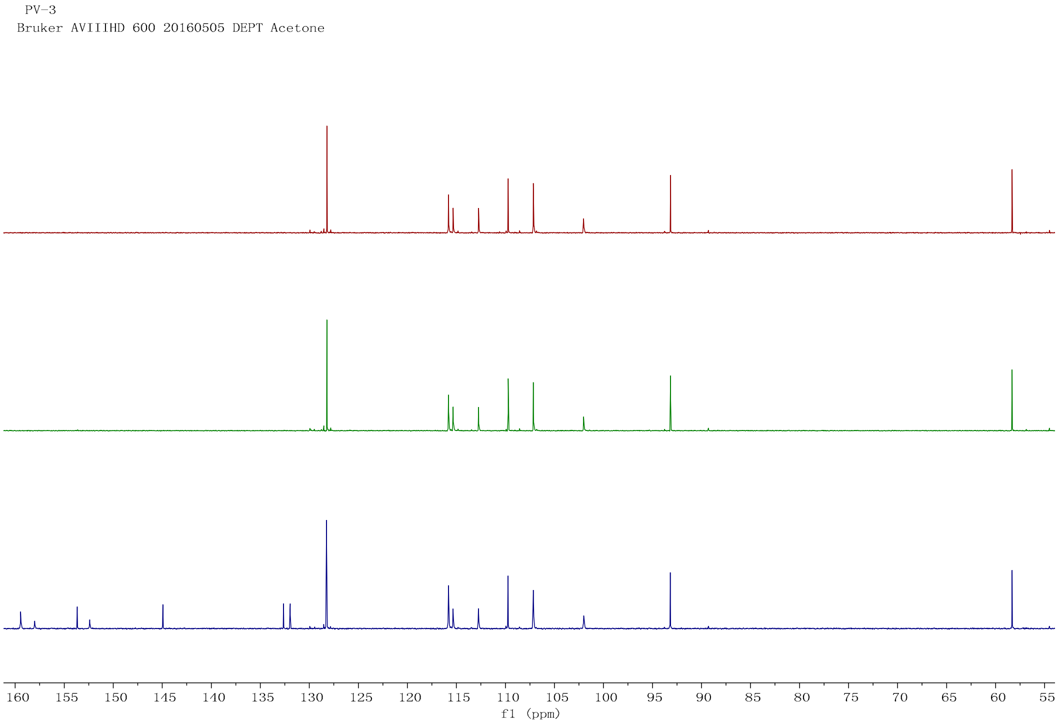

S23. HSQC spectrum of compound **5** in CD_3COCD_3.

PV-3.
Bruker AVIIIHD 600 20160505 HSQC Acetone

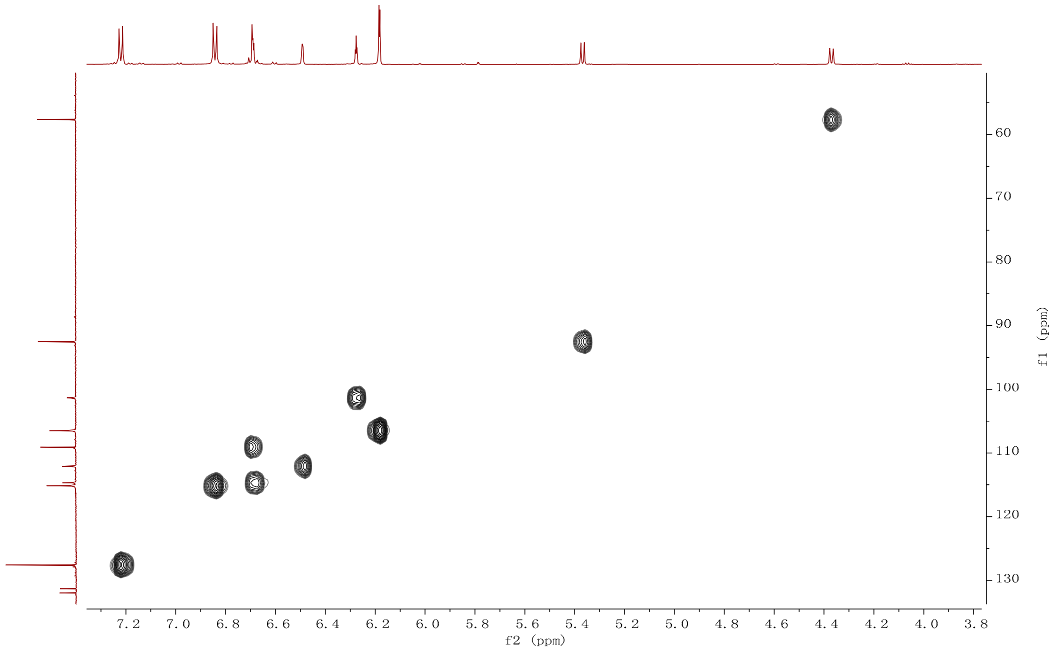

S24. HMBC spectrum of compound **5** in CD_3COCD_3.

PV-3.
Bruker AVIIIHD 600 20160505 HMBC Acetone

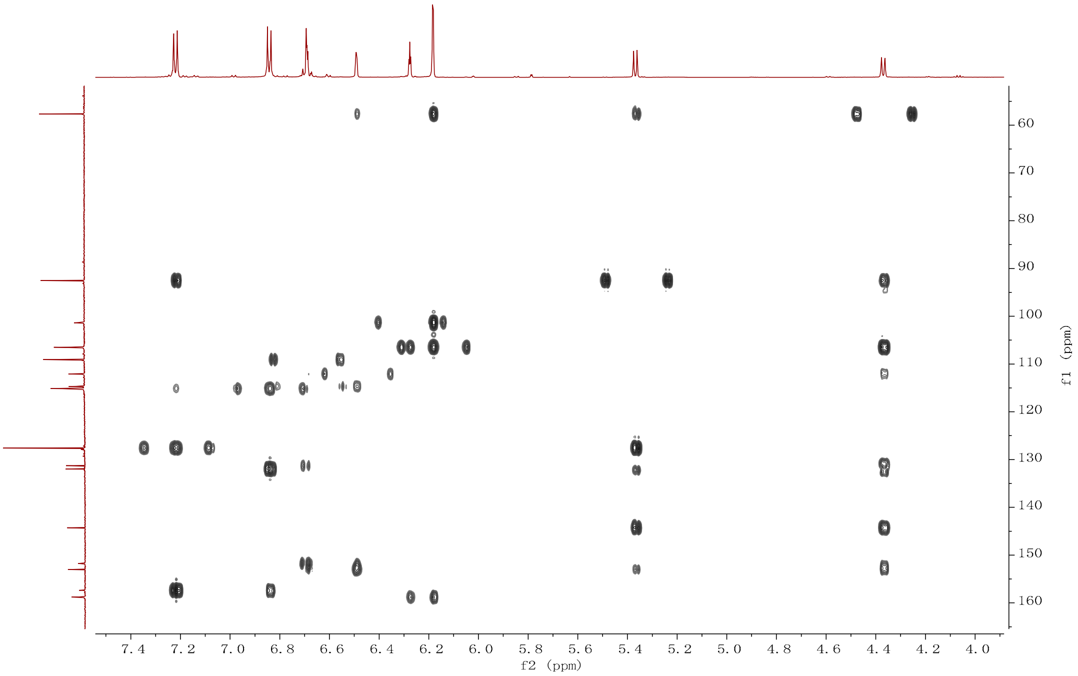

S25. NOESY spectrum of compound **5** in CD_3COCD_3.

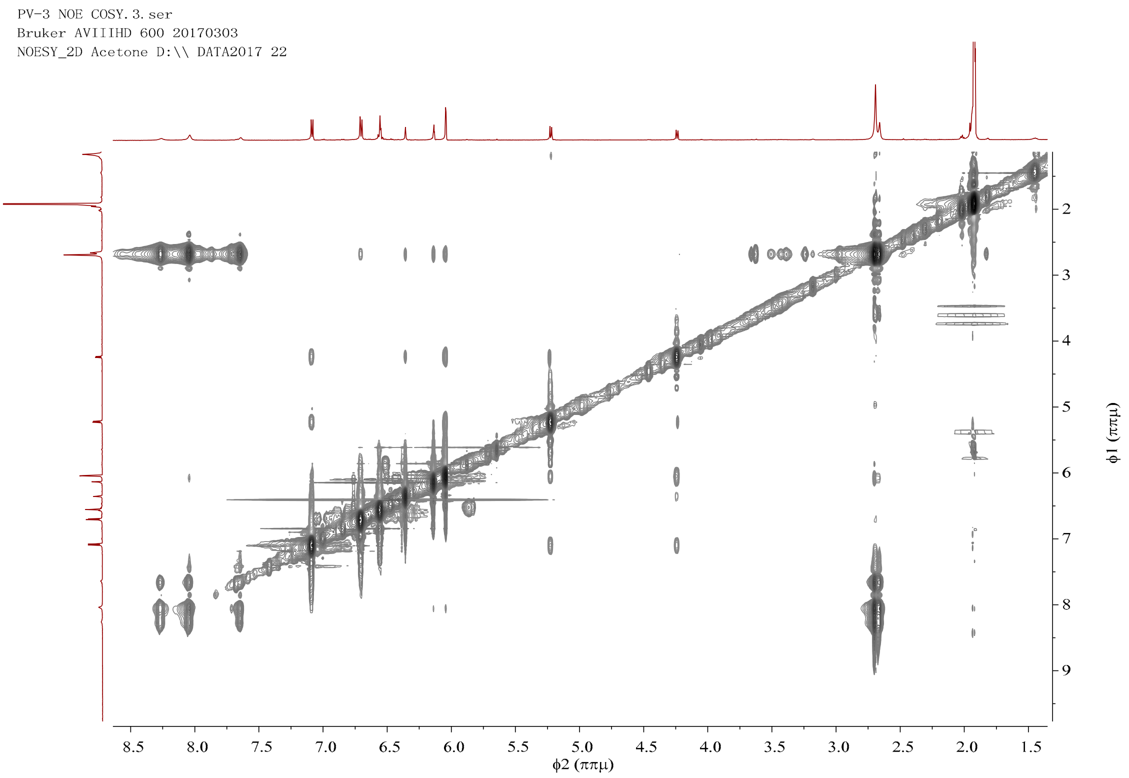

S26. COSY spectrum of compound **5** in CD_3COCD_3.

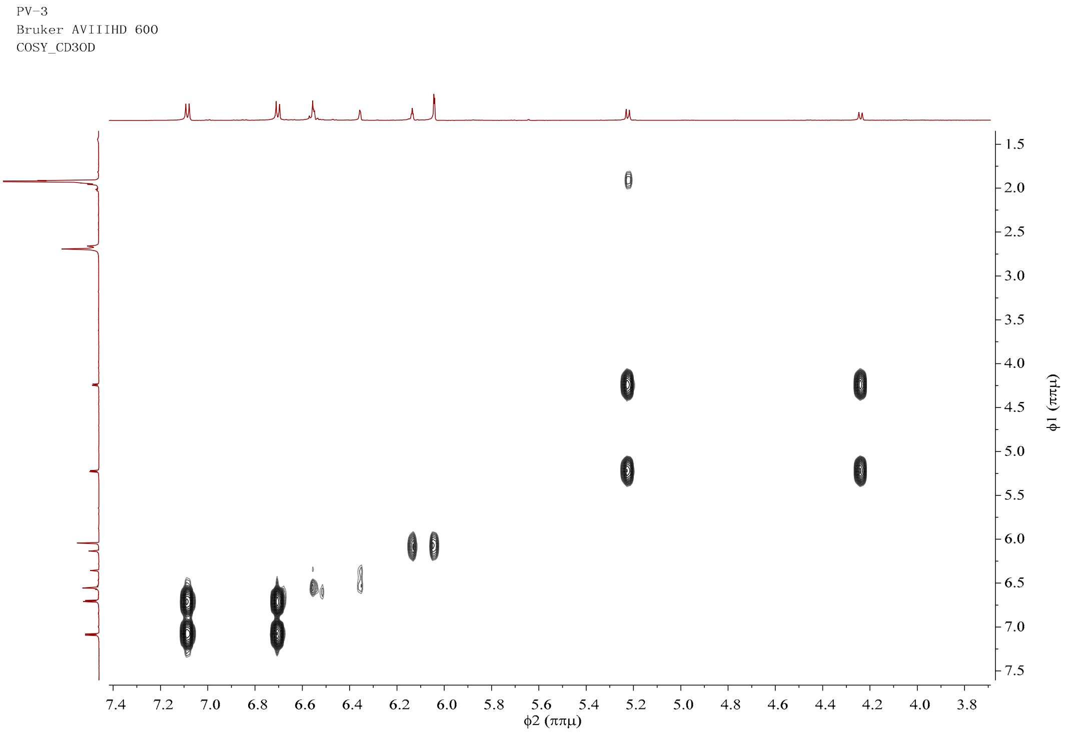

S27. HRESIMS spectrum of compound **5**.

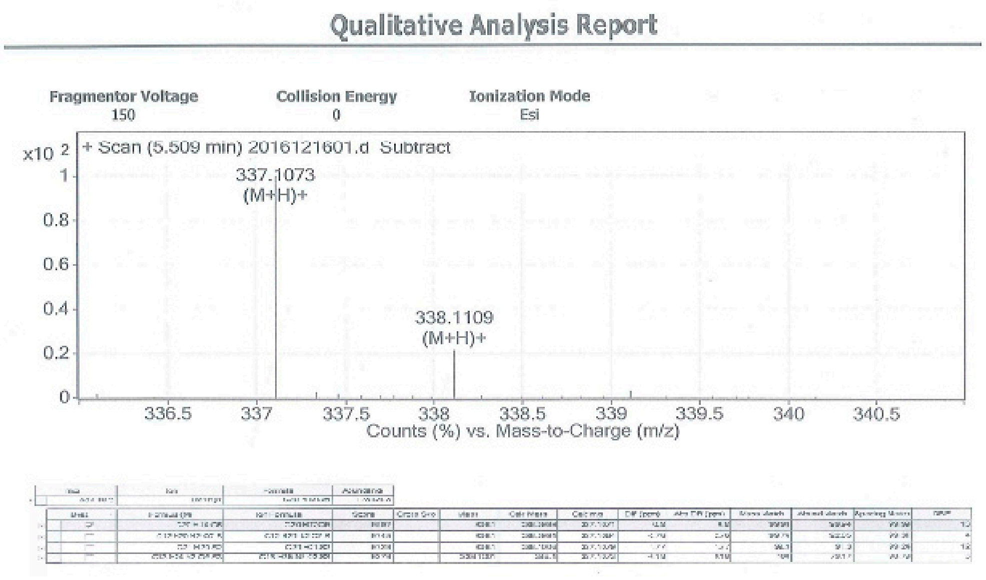

S28. UV spectrum of compound **5** in CH_3OH.

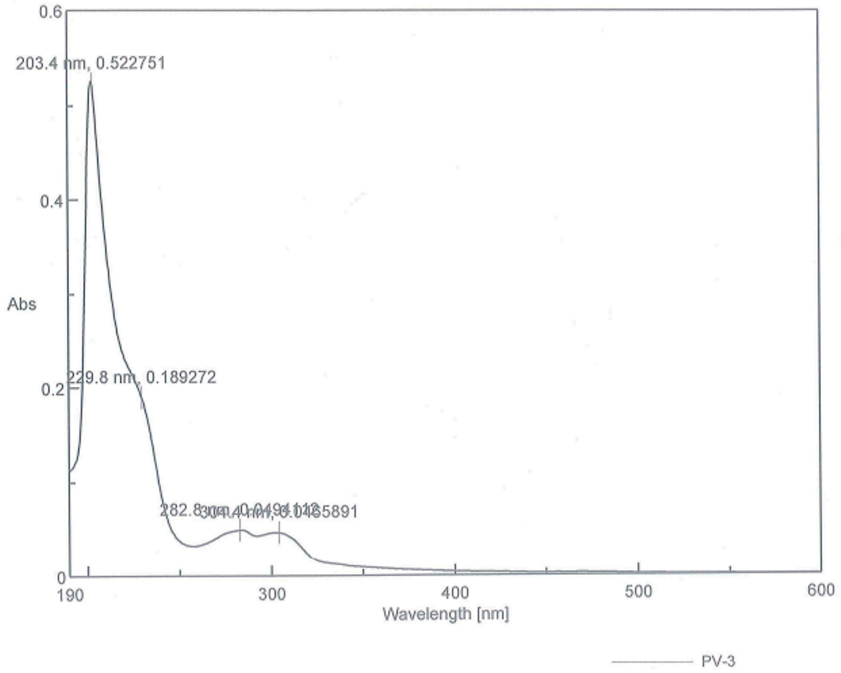

S29. IR spectrum of compound **5**.

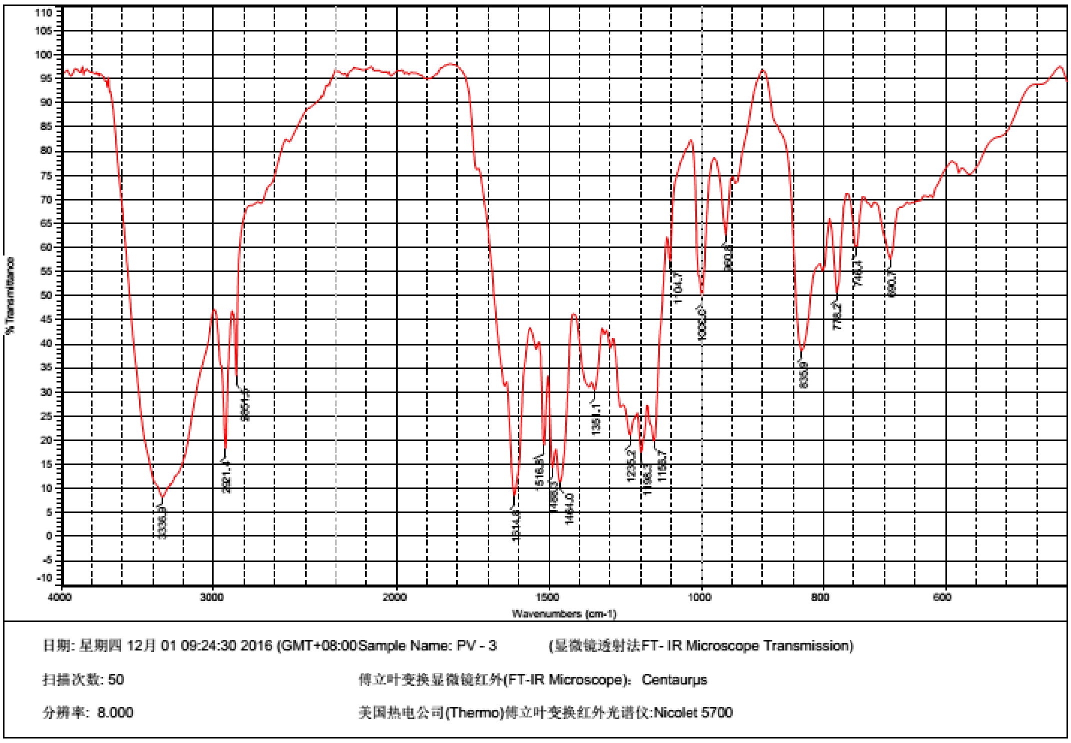

S30. HPLC chromatogram of biotransformation products of **1** and **2**.

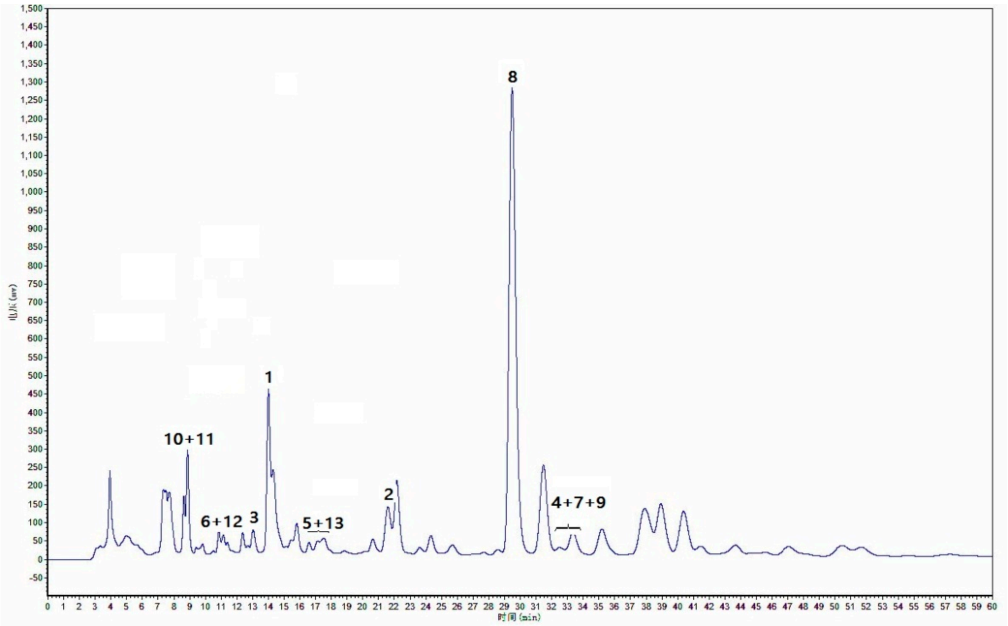

Analysis of biotransformation products of **1** and **2** catalyzed by HRP/H_2O_2.

(40% CH_3CN/H_2O, λ = 280 nm, 2 mL/min, YMC column packed with C18, 250 mm × 10 mm)
